# Ethnic-cultural procedural fairness effects on organizational identification and job satisfaction among minority and majority employees

**DOI:** 10.3389/fpsyg.2025.1445469

**Published:** 2025-04-15

**Authors:** Kim Dierckx, Hilde Depauw, Tessa Haesevoets, Barbara Valcke, Thomas Van Roey, Bart Van de Putte, David De Cremer, Crizelle Els, Alain Van Hiel

**Affiliations:** ^1^Department of Developmental, Personality, and Social Psychology, Faculty of Psychology and Educational Sciences, Ghent University, Ghent, Belgium; ^2^Department of Sociology, Faculty of Political and Social Sciences, Ghent University, Ghent, Belgium; ^3^D’Amore-McKim School of Business, Northeastern University, Boston, MA, United States; ^4^Workwell Research Unit, North-West University, Potchefstroom, South Africa

**Keywords:** ethnic-cultural procedural fairness, collective procedural fairness (CPF) model, diversity management, organizational identification, job satisfaction

## Abstract

In the present contribution, we examined the application of procedural fairness in the resolution of ethnic-cultural (EC) issues, which are issues relating to ethnic, cultural, and linguistic matters. We hypothesized that EC procedural fairness perceptions contribute to effective diversity management because they are positively related to job satisfaction among minority group employees. We further theorized that this relationship is mediated by organizational identification. What makes the present study particularly unique is that we employ a dual focus, by examining the perceptions of both minority and majority group members. Two field studies (total *N* = 2,059; 26.3% minority members) and a longitudinal field survey (*N* = 265 minority members) supported our predictions. In Study 1, we consistently found that minority employees’ EC procedural fairness perceptions were positively associated with job satisfaction. Moreover, organizational identification fully mediated this relationship. Interestingly, similar positive responses to EC procedural fairness were observed among majority group employees. Study 2 sampled minority employees working in various countries and industrial sectors on two different measurement occasions. Multilevel mediation analyses provided further support for the mediating role of organizational identification. Finally, Study 3 sampled minority and majority group assembly line workers pertaining to various ethnically diverse teams. In line with Study 1, our multilevel analyses revealed that EC procedural fairness perceptions were related to enhanced job satisfaction (through organizational identification) among minority *and* majority group employees. Taken together, the present results highlight that procedural fairness can be implemented to resolve ethnic-cultural issues in today’s super-diverse organizations, and by doing so, they emphasize the potential of procedural fairness for organizational diversity management.

## 1 Introduction

Globalization—i.e., the growing interconnectedness of the world’s economies, cultures, and populations—has had a tremendous impact on the 21st century labor market, and in particular on the ethnic-cultural composition of the contemporary workforce (Kharroubi, 2021). According to recent estimates, international migrants account for 4.7% of the total global labor force (International Labour Organization, 2024), and these numbers are expected to increase over the coming decades (De Jager, 2024). At the same time, demographic within-country changes have further accelerated the diversification process in organizations, transforming companies into truly multicultural work environments that bring together individuals from diverse backgrounds and perspectives (Farnsworth et al., 2024).

As a direct consequence of their rapidly diversifying workforces, management and team supervisors need to increasingly operate as company “diversity managers” (Homan et al., 2020), in order to reap the benefits—and not the possible hurdles—associated with diversification. In this regard, Guillaume et al. (2014) have proposed that factors promoting an “inclusive work environment” are key to the successful and effective management of organizational diversity. Specifically, in their multilevel model of diversity management, these authors highlight the pivotal role of organizational policies and procedures, and they argue that “the extent to which these [policies and procedures] facilitate the integration of differences, lead to equitable employment practices, and promote the inclusion of all employees in decision making… will promote [minority] employee wellbeing” (Guillaume et al., 2014, p. 787). Bearing this in mind, it can be expected that a particularly critical feature of diversity management will become how organizational decision-makers deal with ethnic-cultural (EC) issues. EC issues are issues relating to ethnic, cultural, and linguistic matters.

As an example, imagine a situation whereby a team supervisor must determine whether to allow prayers during work hours or not. Or, in a large and diverse organization, the management may be obliged to consider incorporating the dietary preferences of religious subgroups into their company cafeteria menu (e.g., serving of kosher or halal food), or allowing members of linguistic subgroups to communicate in their home language with one another (e.g., to facilitate the explanation and comprehension of workplace protocol). In all the above cases, the interests of one or more specific ethnic-cultural groups are at stake (e.g., those who profess a given religion, in the first two examples). It thus stands to reason that the way organizational decision-makers resolve these issues can foster (or undermine) the perception of an inclusive work environment among EC minority group employees.

**Figure 1:**
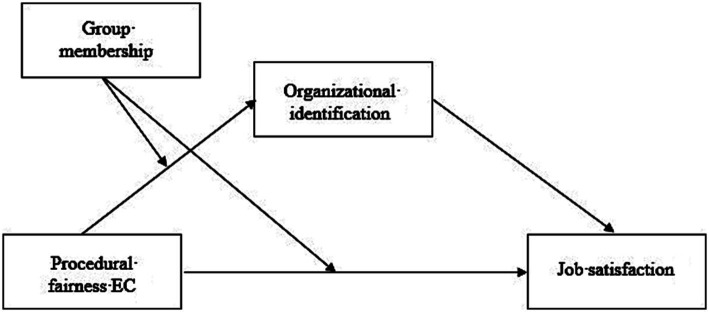
Schematic overview of the hypothesized relationship between EC procedural fairness perceptions, organizational identification and job satisfaction.

In line with the above critical predictions of Guillaume et al.’s (2014) model, the present contribution aims to answer the following central question: *Can decision-makers in diverse organizations promote such a climate of inclusion by applying the principles of procedural fairness when resolving EC issues?* Procedural fairness (or procedural justice) refers to the extent to which decisions are made fairly, consistently, and without bias (Leventhal, 1980; Lind and Tyler, 1988; Tyler, 1988; Van den Bos et al., 2001). Building on Collective Procedural Fairness theory (CPF; Dierckx et al., 2023a; Valcke et al., 2020a, 2020b), we develop the argument that EC procedural fairness perceptions—which we define as the perception that decisions vis-à-vis EC issues are generally made in a procedurally fair way—enhance minority employees’ organizational identification, which, in turn, has positive downstream consequences for their job satisfaction. Although a few studies have documented the usefulness of implementing procedural fairness in EC issues (e.g., Peate et al., 2008; Platow et al., 1998, 2008, 2013), we are not aware of any research aimed at characterizing EC procedural fairness effects in organizational contexts. Therefore, to tackle this lacuna in literature, the central aim of the present study is to explore the usefulness of the implementation of procedural fairness in the resolution of organizational EC issues. Specifically, we investigate the relation between minority employees’ EC procedural fairness perceptions and their organizational wellbeing (i.e., organizational identification, job satisfaction). We further verify that these relationships materialize while controlling for “general”^1^ (non-cultural) procedural fairness perceptions. Moreover, besides focusing on the minority perspective, we additionally investigate majority group employees’ reactions to the perception of EC procedural fairness, and directly compare them with the minority perspective. By doing so, we integrate the social-psychological CPF model (Dierckx et al., 2023a; Valcke et al., 2020a, 2020b) into Guillaume et al.’s (2014) organizational model of diversity management, as we show that EC procedural fairness can contribute to the effective management of a diverse workforce. Figure 1 presents the conceptual model of our study.

### 1.1 Theoretical background and hypotheses development

To the best of our knowledge, only a few seminal studies have thus far examined if procedural fairness can be incorporated into the decision-making process surrounding EC issues and how this affects the reactions of those involved. Platow et al. (2008) and Peate et al. (2008) focused their attention on how authorities dealt with real-life intergroup disputes in the Australian context (e.g., riots between ethnic groups in Sydney, settlements of land claims by the traditional Australian community and non-Indigenous Australians). These seminal studies revealed that the perception of procedural fairness vis-à-vis the resolution of the EC issues under scope increased acceptance of and decreased protests against authorities’ final decisions. Relatedly, experimental work by Platow et al. (2013) (Study 2) demonstrated that voice provision to participants (in comparison to voice denial) resulted in more positive evaluations of the leader dealing with the EC issue. In sum, it thus appears that procedural fairness is a very relevant concept for EC decision-making. In the current research, however, we argue that EC procedural fairness effects can extend beyond sheer evaluations of aspects of the decision-making process (e.g., evaluations of outcomes, authority figures, etc.) and impact upon the organizational wellbeing of the minority workforce.

To ground our theoretical predictions, we draw on the work of Valcke et al. (2020a, 2020b), who investigated the effects of EC procedural fairness enactment by societal decision-makers (i.e., the courts, the police, political stakeholders). In brief, these scholars consistently observed a positive relationship between minority group members’ perceptions of procedurally fair treatment by societal actors and their wellbeing (i.e., their experienced life satisfaction and social wellbeing). To explain their findings, Valcke et al. (2020a, 2020b) developed a theoretical model which grounds these positive reactions to group-based fair treatment in processes of self-categorization (Turner et al., 1987; Turner et al., 1987). Specifically, the CPF model (Dierckx et al., 2024; Valcke et al., 2020a, 2020b) holds that EC procedural fairness calls attention to the fact that oneself is a member of the fairness beneficiary minority group—or a member of a minority group in general (see Dierckx et al., 2023b)—which initiates a shift in self-perception from personal to social identity, spurring members of the involved minority group to temporarily redefine themselves in terms of their minority group membership. Once this perceptual shift has occurred, the message of acceptance conveyed by the fair treatment of minority groups naturally extends to the individual minority group member (Dierckx et al., 2024). In other words, according to the CPF model, EC procedural fairness does not only constitute a collective token of appreciation, but it also implicitly conveys the message that the individual minority citizen—being a member of an EC group—is valued by the particular decision-maker (Valcke et al., 2020a, 2020b). And this message of valorization and acceptance, in turn, positively impacts minority group members’ experienced wellbeing.

### 1.2 Existing evidence for the distinction between EC and “general,” non-EC procedural fairness

Importantly, the emerging literature on EC procedural fairness suggests that fairness evaluations vis-à-vis EC issues differ from fairness judgments related to non-EC matters in conceptually meaningful ways. Firstly and most importantly, research has revealed that distinct psychological processes underly each type of fairness perceptions. Specifically, general procedural fairness effects have been shown to be regulated by employees’ *relational* self-concepts, and specifically by those aspects of the self that they derive from the relationship with the fairness-enacting authority (Lind and Tyler, 1988; Tyler and Blader, 2000; Tyler and Lind, 1992). By contrast, Dierckx et al. (2023b, 2024) and Valcke et al. (2020a, 2020b) have revealed that EC procedural fairness primarily enhances the saliency of one’s EC group membership, and thus activates psychological processes that operate at the *collective* levels of the social self.

Secondly, research further demonstrates that general and EC procedural fairness tap into different human needs. That is, one of the key premises of the relational models of procedural justice (Lind and Tyler, 1988) is that people care about fair treatment by authorities because it satisfies their relational needs, i.e., the personal need to belong to a group or multiple groups (Baumeister and Leary, 1995). In line with this assertion, reactions to general procedural fairness tend to be particularly positive among those with a high need to belong (e.g., De Cremer and Blader, 2006). However, it should be emphasized that people can also have needs on behalf of their social groups—e.g., they can desire that their group is accepted and valued in society—and it is exactly these “collective needs” which EC procedural fairness has been found to fulfill. For example, EC procedural fairness perceptions can enhance societal belongingness among EC minorities (i.e., their sense of belonging and commitment to society at large; Valcke et al., 2020a) and reduce perceptions of ingroup-directed discrimination (Dierckx et al., 2021)—thereby satisfying their need for “group relatedness” (Kachanoff et al., 2020). Thus, general and EC procedural fairness are each related to the satisfaction of different psychological needs, which further corroborates the notion that they can conceptually—and empirically—be discerned.

Third, general (procedural, but also distributive) fairness has mainly been related to within-group cohesion (e.g., team commitment, relations between members of the same work unit; Colquitt et al., 2023). By contrast, EC procedural fairness has been associated with enhanced intergroup relations (e.g., Dierckx et al., 2020b). Thus, it appears that both types of fairness differ in their importance or their potential contribution to organizational cohesion: Whereas general fairness enactment can serve to facilitate intragroup *bonding* (i.e., between members of the same team, ethnicity, etc.), EC fairness can be implemented to promote intergroup *bridging* (i.e., between majority and minority group members, between members of different minority groups, etc.). EC fairness is thus not just another form of procedural fairness; it carries unique significance for fostering organizational harmony across ethnic and cultural boundaries. In sum, in light of the evidence reviewed above, in the current study we will treat EC and general procedural fairness perceptions as two different (but related) constructs, which can each have meaningful downstream organizational consequences, above and beyond each other (but note that we will additionally highlight evidence in our data that supports this conceptual distinction in the organizational context). Two particularly relevant organizational outcomes are discussed in the next sections, those being: job satisfaction and organizational identification.

### 1.3 EC procedural fairness and minority employees’ job satisfaction

Applying the CPF model to the organizational setting, we argue that the message of acceptance and inclusion conveyed by procedural fairness enactment in EC decision-making should be positively related to job satisfaction among minority employees. Job satisfaction is broadly defined as “the positive emotional state that one derives from experiences associated with one’s job” (Fernández-Macias and de Bustillo Llorente, 2023). Indeed, there are good theoretical reasons to expect a positive EC procedural fairness-job satisfaction association. First of all, ample research has shown that granting people voice, i.e., giving them the opportunity to express their views and opinions, increases the perceived fairness of a given decision procedure, and hence, positively impacts their organizational wellbeing (Colquitt et al., 2023; Schmitt and Dörfel, 1999). However, it can be expected that, for EC minorities, voice opportunities are even more pivotal than for their majority counterparts. That is, because of their minority status, members of minoritized groups are less likely to perceive decision control in organizational issues. As such, knowing that they can provide input and be confident that their views will be taken into account should elicit positive reactions among members of groups that are minoritized in numbers, because it increases their perception of successful influence over organizational decisions. Moreover, voice can also have strong symbolic value for EC minority group members because it “reaffirms an interest in their opinions and consequently increases their feelings of self-esteem and feelings of inclusion” (Hunton et al., 1996). For both reasons, it stands to reason that granting minority employees voice opportunities, in particular when it comes to issues that concern them, may have a positive impact on the way they feel in their job and organization.

In a related vein, research has further shown that EC minorities are, due to their elevated exposure to discrimination, highly sensitive to cues of discriminatory treatment (Kaiser et al., 2006). Consequently, EC minority members may pay particular attention to whether or not organizational decision-makers implement unbiased and impartial decision procedures. It can thus reasonably be expected that the procedurally fair resolution of EC issues—which, by definition, fosters the impression of a bias-free work environment—should boost the organizational wellbeing of EC minority employees. Lastly, various studies have shown that minority employees attach substantial importance to diversity management (e.g., Farashah et al., 2024), which is reflected in the observation that they report higher job satisfaction in the presence of (effective) diversity management policies (Pitts, 2009). It therefore stands to reason that, to the extent that they perceive the implementation of inclusive procedures to be a manifestation of effective diversity management, procedurally fair treatment regarding EC issues should analogously be reciprocated with higher job satisfaction among the minority workforce. Based on the above theoretical arguments, we thus formulate the following hypothesis:

*Hypothesis 1:* EC procedural fairness perceptions are positively related to job satisfaction among minority employees.

### 1.4 Organizational identification as an explanatory process variable

To explain the hypothesized relationship between EC procedural fairness and minority employees’ job satisfaction, we connect the CPF model (Dierckx et al., 2024; Valcke et al., 2020a, 2020b) with Guillaume et al.’s (2014) model of diversity management. The main assumption of the CPF model is that the experience of EC procedural fairness “initiates a process of perceptual categorization, whereby minority group members temporarily redefine themselves in terms of their minority group membership” (Dierckx et al., 2024, p. 12). Importantly, Valcke et al. (2020a, 2020b) further assert that this passive and transient form of self-definition as an EC minority group member paves the way for more active, affective, fairness-induced forms of identification with superordinate categories. The latter conjecture resonates well with the reflections of Guillaume et al. (2014), who contend that “effective diversity management procedures… that are implemented by supervisors at the work group level… address [minority] employees’ identity concerns^2^” (p. 787). In other words, these authors thus advance the hypothesis that inclusion-promoting interventions—such as EC procedural fairness enactment—are effective because they increase minority group members’ organizational identification, defined as “the extent to which an individual identifies with and has feelings of loyalty toward his/her work organization” (Blader et al., 2017, p. 20). In line with this assertion, the perception that one’s organization acknowledges the value of diversity through the procedures they enact (i.e., a “psychological diversity climate”) has been shown to relate strongly and positively to minority employees’ organizational identification (Cole et al., 2016). In a similar vein, an inclusive decision-making style, which “promotes the inclusion of all team members” (Mitchell et al., 2015, p. 218), and entails that leaders “commit to ensuring all team members are treated equitably” (Center for Creative Leadership, 2022) has been associated with organizational identification (Yasin et al., 2023; Shore and Chung, 2022). These findings thus corroborate our contention that EC procedural fairness perceptions are positively related to organizational identification.

Moreover, Guillaume et al.’s (2014) diversity management model further holds that enhanced identification and commitment will lead to more favorable work-related outcomes in diverse organizations. And indeed, enhanced organizational identification has been associated with a plethora of indicators of wellbeing at work (see Weisman et al., 2023, for a review), including job satisfaction (e.g., De Giorgio et al., 2023). Hence, these theoretical reflections and the associated empirical evidence supportive of it lead us to hypothesize that the expected positive relationship between EC procedural fairness and job satisfaction is mediated by organizational identification. As such, we formulated the following hypothesis:

*Hypothesis 2:* The relationship between EC procedural fairness perceptions and job satisfaction is mediated by organizational identification among minority employees.

### 1.5 Majority perspective on ethnic-cultural procedural fairness enactment

Although the studies identified by our literature review thus far seem to paint a rather promising picture with regard to the potential of EC procedural fairness for diversity management, it is also important to identify potential caveats and pitfalls, in order to create a more holistic understanding of what EC procedural fairness can and cannot do for diverse organizations. For example, prior research (in non-organizational settings) has already cautioned against *sedative* effects of procedurally fair treatment of minority groups. Specifically, Dierckx et al. (2023a) showed that EC procedural fairness perceptions can reduce minority group members’ attention to the structural disadvantages they suffer, which in turn curbs their support for collective actions that could enhance their social status. In a related vein, another study has shown that procedural fairness enacted by societal actors (e.g., the courts, politicians) may strengthen the bond between minority group members and society’s institutions, while at the same time it can unintentionally spark intergroup tensions among minority groups (Dierckx et al., 2020a).

In the light of this last finding, an important final question to add to our research is thus: How does the majority group workforce react to EC procedural fairness enactment? The relevant literature suggests two mutually exclusive possibilities. First, it might be that the perception of EC procedural fairness affects majority members negatively. According to Realistic Group Conflict Theory (Sherif, 1966), groups tend to compete for limited resources, and organizational procedurally fair treatment can be considered such a “resource” (Cropanzano et al., 2001) that will become scarcer when already displayed to others (Camps et al., 2019). From this perspective, majority group members may conceive of EC procedural fairness as conflicting with fairness enactment in domains that serve their own goals and interests, and they may perceive themselves to be “on the losing end” (see for example Brown and Jacoby-Senghor, 2021, in this regard). Additionally, the implementation of EC procedural fairness may increase the perception of decision control by minority group employees (Thibault and Walker, 1975), and may therefore be interpreted as hurting the majority’s interests, which could lead to their disapproval. For both reasons, EC procedural fairness perceptions may thus negatively impact upon organizational identification and job satisfaction among majority employees, which leads to the following prediction:

*Hypothesis 3a:* The direct relationship between EC procedural fairness perceptions and job satisfaction, as well as the indirect relationship (through organizational identification) are moderated by group membership, such that positive effects are observed among minority group employees and negative effects among majority group employees

Conversely, the deontic model of fairness proposes that people care about justice “as such, out of respect for humanity” (Folger, 2001, p. 7). Deontic theory further states that universal morality-based justice concerns play a role in the perception of third parties, which may prompt them to display negative attitudes toward injustices experienced by others, and conversely, foster positive attitudes toward authorities enacting fairness vis-à-vis others (Beugré, 2012; Folger, 2001; Zwank et al., 2024). The deontic model would thus predict that corporate social responsibility in the form of EC sensitive and inclusive procedures may actually benefit fairness perceptions, and in turn, organizational identification and job satisfaction among the majority group (Dunford et al., 2014). As such, based on the deontic account (Folger, 2001), we formulate the prediction that EC procedural fairness perceptions may also be positively associated with organizational identification and job satisfaction among majority employees:

*Hypothesis 3b:* The direct and indirect relationship between EC procedural fairness perceptions and job satisfaction are not moderated by group membership; such that the positive effects of procedural fairness are similar for minority and majority employees.

## 2 The present studies

In sum, the central goal of the present research was thus to investigate if procedural fairness can be implemented to deal with EC issues in organizational settings. Building on Guillaume et al. (2014) and the work of Dierckx et al. (2023a) and Valcke et al. (2020a, 2020b), we hypothesized that (1) EC procedural fairness perceptions are positively related to job satisfaction among minority employees, and (2) that this relationship is mediated by enhanced organizational identification. In addition, we gauged the reactions of majority group members to EC procedurally fair treatment and assessed if they differed from those observed among the minority beneficiaries. To test our hypotheses, we conducted two field studies (Studies 1 and 3) and one longitudinal field study (Study 2). Study 1 recorded employees’ EC fairness perceptions in three different samples [blue-collar logistics employees in a Western European multinational (Sample 1a), blue and white-collar workers in a governmental company (Sample 1b), and blue-collar employees in a moderately sized service company (Sample 1c)], to test our basic mediation model. Samples 1b and 1c also gauged general, non-EC fairness perceptions, to assess whether EC fairness explains unique variance in the outcome variables. Furthermore, Sample 1c focused on a *specific* EC decision (i.e., about language policy) rather than employees’ perceptions of how EC decisions are *generally* made. In Study 2, we sampled Muslim minority group workers from various countries and organizations, and we probed their EC procedural fairness perceptions, organizational identification, and job satisfaction on two different measurement occasions. Finally, Study 3 measured EC and general fairness climate perceptions among blue-collar workers pertaining to more than 100 ethnically diverse work teams within another Western European multinational company.

It is important to note that our studies complement each other, as each study addressed specific research questions emerging from the previous one(s). Study 1 attempted to explore the basic (cross-sectional) relations encapsulated in our model among minority and majority workers. Study 2 built on Study 1 because it gauged how these relationships evolve over time and as such, it aimed to provide additional support for the directional nature of the hypothesized associations among the focal variables. Finally, Study 3 provided a multi-level analysis of how the Study 1 and Study 2 results vary as a function of team dynamics.

All data, analysis code, and research materials are available at our Open Science webpage https://osf.io/7an8d/. An in-depth description of all covariate analyses can also be found there (document “Covariate Analyses”), as well as all Supplementary materials referred to below (e.g., tables, etc.—see document “Online Appendix”).

## 3 Study 1

### 3.1 Participants

#### 3.1.1 Sample 1a

A total of *N* = 801 logistics employees pertaining to three company sites completed our survey (203 minority group members;^3^ age: *M* = 36.98*, SD* = 10.63, range = 18–69; 47.8% males). Prospective participants were approached by the researchers at the beginning of their shifts or during lunchbreak. Surveys were completed in paper-pencil format or online.

#### 3.1.2 Sample 1b

A total of *N* = 514 governmental employees completed our online survey (78 minority group members; age: *M* = 41.68*, SD* = 10.27, range = 21–64; 66.0% males). Participants were recruited online, by means of an email sent out to the entire company.

#### 3.1.3 Sample 1c

A total of *N* = 235 employees, working in a medium sized company which provides house-to-house cleaning services for private costumers, completed our paper-and-pencil survey (51 minority group members; age: *M* = 45.36, *SD* = 10.08, range = 21–64; 1.0% males). Participants were recruited during bimonthly meetings with their job coaches, and surveys were completed in paper-pencil format or online.

#### 3.1.4 Power sensitivity analyses

Given that sample sizes were based on availability of voluntary participants, we conducted a series of power sensitivity analyses to calculate the minimum detectable effect size (*α* = 0.05). Results revealed that our studies had 80% power to detect minimal slopes for our mediator of size *b* = 0.09, *b* = 0.10 and *b* = 0.16, in samples 1a–1c, respectively.

### 3.2 Measures

All items were embedded in larger surveys on diversity in the workplace. To maximize the response rate, the surveys were deliberately kept short. Consequently, the number of items to measure each construct was significantly reduced, in comparison to the full scales.

We measured *EC procedural fairness* with six items (scaled 1 = completely disagree, 5 = completely agree) adapted from Valcke et al. (2020a, 2020b). These items were based on Naumann and Bennett’s (2000) measure, and the adapted scale was rigorously validated in Valcke et al. (2020a, 2020b). These items were preceded by an introductory sentence:

*“At [company], the workforce consists of people belonging to a* var*iety of cultures, ethnicities, and religions… And sometimes, decisions need to be made which affect an entire group of people belonging to the same ethnic-cultural group; How are, generally speaking, such decisions made according to you?”*^4^

A few examples of decisions that relate to such issues were also given (e.g., the decision to allow prayers at work, or the decision to customize the cafeteria menu in order to include kosher and/or halal food). A sample item is “Accurate information is used to make such decisions” (Sample 1a: *α* = 0.85; *M* = 3.46, *SD* = 0.75; Sample 1b: *α* = 0.85; *M* = 3.06, *SD* = 0.71; Sample 1c: *α* = 0.72; *M* = 3.91, *SD* = 0.61). Furthermore, in Samples 1b and 1c, *general (non-EC) procedural fairness perceptions* were also recorded. In Sample 1b, we measured this variable with five out of the six items used to measure EC procedural fairness^5^ (*α* = 0.77; *M* = 2.75, *SD* = 0.73). In Sample 1c, we measured this variable with one item assessing one’s team leader’s decision-making (i.e., “When my team leader makes decisions that concern me, he/she does it in a fair way”; *M* = 4.17, *SD* = 0.80).

To measure *organizational identification*, we selected two^6^ items from Leach et al. (2008) which yielded the best psychometric properties across two pilot studies (i.e., loadings of >0.95) and displayed the expected theoretical relationships with key criterion variables, i.e., “I feel strongly committed to [company]” and “I am proud to work for [company]” (scaled 1 = completely disagree, 5 = completely agree) (Sample 1a: *α* = 0.89; *M* = 4.05, *SD* = 0.85; Sample 1b: *α* = 0.85; *M* = 3.74, *SD* = 0.84; Sample 1c: *α* = 0.84; *M* = 4.06, *SD* = 0.89). We deliberately included items tapping into the core dimensions of *self-investment*, and not *self-definition*, to discern our psychological mediator from the early perceptual self-categorization processes which have been shown to initiate EC procedural fairness effects (see Dierckx et al., 2023b, 2024, for a similar approach). It should further be noted that we implemented the full Leach et al. (2008) scale in Study 2, and the main results and key relationships were virtually identical in that study.

*Job satisfaction* was measured by a single item: “How satisfied are you with your current job” (scaled 1 = very dissatisfied to 5 = very dissatisfied, Sample 1a: *M* = 3.95, *SD* = 0.88; Sample 1b: *M* = 3.76, *SD* = 0.93; Sample 1c: *M* = 4.23, *SD* = 0.86).

Supplementary Table A2 further provides an exhaustive overview of the study-specific covariates, and the items used to measure them.

### 3.3 Data-analysis and results

We first analyzed the data of the minority employees to investigate Hypotheses 1–2.

#### 3.3.1 Correlation analysis

Supplementary Tables B1–B3 display the correlations matrices, broken down by group membership. A closer look at these tables reveals that EC procedural fairness and job satisfaction were positively and significantly associated in all samples (*r*s ranging from 0.28–0.48, all *p*s < 0.001). These findings thus provide clear evidence for our prediction that, among minority members, procedural fairness perceptions concerning EC decision-making are positively related to job satisfaction (i.e., *Hypothesis 1*).

#### 3.3.2 EC vs. general procedural fairness: confirmatory factor analyses

As an initial data check, we verified whether EC and general procedural fairness perceptions could indeed empirically be distinguished (as in prior research conducted in non-organizational settings). To do so, we ran two types of analyses for the two samples wherein general procedural fairness perceptions were included (i.e., Samples 1b-1c): (1) a Confirmatory Factor Analysis (CFA), to test whether these fairness measures indeed loaded on different latent factors; and (2) a regression analysis, with both fairness variables as independent variables and organizational identification and job satisfaction as the outcomes, to verify that EC fairness perceptions contributed to the focal outcomes, above and beyond general fairness perceptions.

The results of our CFAs are reported in detail in the Supplementary Tables C1, C12. For both samples, it was shown that the two-factor solution models significantly outperformed models with a single, latent fairness factor (Sample 1b: fit two-factor solution = *χ^2^*(25) = 60.57, *p* < 0.001, *χ^2^/df* = 2.42, CFI = 0.98, TLI = 0.97, RMSEA = 0.05 [0.03, 0.07], SRMR = 0.03; results of a Likelihood Ratio Test [LRT] comparing both models = *χ^2^* (1) = 240.48, *p* < 0.001; Sample 1c: *χ^2^*(8) = 8.51, *p* = 0.386, *χ^2^/df* = 1.06, CFI = 0.99, TLI = 0.99, RMSEA = 0.02 [0.00, 0.09], SRMR = 0.03; LRT = *χ^2^* (1) = 161.71, *p* < 0.001).

#### 3.3.3 EC vs. general procedural fairness: regression (relative weight) analyses

Given the substantial correlation between EC and general procedural fairness (*r*s > 0.55), traditional regression analysis could be considered inappropriate due to multicollinearity issues. Hence, we used Relative Weight Analysis (RW; Tonidandel and LeBreton, 2015), a technique which “filters out” the variance a predictor shares with the other independent variables, thereby providing more reliable parameter estimates (Tonidandel and LeBreton, 2015).

The results of this analysis for Sample 1b revealed that EC and general procedural fairness were each uniquely and significantly associated with job satisfaction (EC: *b* = 0.07, 95% CI = [0.040, 0.116]; general: *b* = 0.16, 95% CI = [0.102, 0.215]) among minority employees. Analogously, for Sample 1c, the results of this analysis revealed that EC procedural fairness perceptions with respect to the language policy decision (LP) and general procedural fairness perceptions with respect to one’s team leader’s decision-making (TL) were each uniquely and significantly associated with job satisfaction (LP: *b* = 0.01, 95% CI = [0.001, 0.159]; TL: *b* = 0.09, 95% CI = [0.013, 0.236]) among minority employees. Not only do these findings provide further clear evidence for *Hypothesis1*, they also demonstrate that EC and general, non-EC fairness perceptions can indeed empirically be distinguished in organizational settings.

#### 3.3.4 Mediation analysis

**Figure 2:**
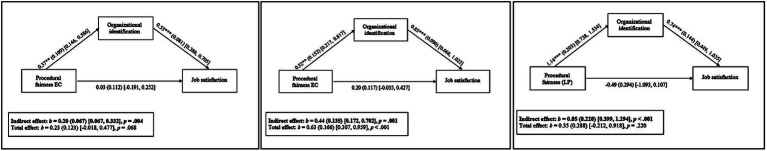
Schematic overview of results of mediation analysis (minority group subsamples, Study 1) with EC procedural fairness as predictor, organizational identification as mediator, and job satisfaction as dependent variable. Notes. *: *p* < 0.05, ** *p* < 0.01, *** *p* < 0.001. Statistically controlled for age, interactional fairness, diversity beliefs, perceived diversity, union membership, work shift (day/night/weekend) and type of employment (fulltime/part time). Reported effects are unstandardized [standard errors in parentheses, and 95% confidence intervals between square brackets]. Left panel = Sample 1a, middle panel = Sample 1b, right panel = Sample 1c.

To investigate *Hypothesis 2*, we fitted the mediation model depicted in Figure 1 for the minority subsample, while controlling for sample-specific covariates.^7^ All analyses were conducted using the *Lavaan* (Rosseel, 2012) and *semTools* (Jorgensen et al., 2018) packages in R (R Core Team, 2021). Prior to our mediation analyses, missing data were imputed using the *mice* package (Van Buuren and Groothuis-Oudshoorn, 2011). In support of *Hypothesis 2*, it was revealed that the relationship between procedural fairness EC and job satisfaction was indeed mediated by organizational identification (Sample 1a: *b* = 0.20, *SE* = 0.067, *p* = 0.004, 95% CI = [0.067, 0.332]; Sample 1b: *b* = 0.43, *SE* = 0.135, *p* = 0.001, 95% CI = [0.172, 0.702]; Sample 1c: *b* = 0.85, *SE* = 0.220, *p* < 0.001, 95% CI = [0.399, 1.294]). Figure 2 (left, middle and right panel) provide overviews of the models and their pathways.

#### 3.3.5 Moderated mediation analysis

Lastly, based on the total samples (including both minority and majority employees) we examined whether EC procedural fairness enactment revealed differential effects for majority and minority members (i.e., *Hypotheses 3a, b*). To this end, we fitted the above mediation model for the full samples, adding both a dummy variable representing group membership (1 = majority group, 0 else), and a second variable encoding the EC procedural fairness × group membership interactions on organizational identification and job satisfaction. The results of this analysis are summarized in Table 1.

**Table 1:** Schematic overview of study-specific moderation analyses: unstandardized betas [95% confidence intervals (CIs) between square brackets], standard errors (*SE*s) and *p*-values are reported.

	PF*Group - ≥ identification			PF*Group - ≥ Job satisfaction			PF*Group - ≥ Identification - ≥ JS		
	*b* [95% CI]	*SE*	*p*	*b* [95% CI]	*SE*	*p*	*b* [95% CI]	*SE*	*p*
1. Study 1, Sample 1a	−0.05 [−0.16, 0.06]	0.05	0.363	−0.00 [−0.11, 0.11]	0.05	0.982	−0.02 [−0.07, 0.03]	0.02	0.351
2. Study 1, Sample 1b	0.06 [−0.08, 0.20]	0.07	0.420	−0.00 [−0.06, 0.05]	0.03	0.880	0.04 [−0.06, 0.15]	0.05	0.416
3. Study 1, Sample 1c	−0.17 [−0.33, −0.00]	0.08	**0.048**	0.06 [−0.02, 0.13]	0.04	0.119	−0.10 [−0.20, 0.00]	0.05	0.053
4. Study 3	−0.03 [−0.13, 0.07]	0.05	0.522	0.01 [−0.03, 0.05]	0.02	0.636	−0.19 [−3.46, 3.09]	1.63	0.909

PF*Group - > Identification: Represents the interaction effect between procedural fairness EC and group membership (majority group vs minority group; the minority group was used as the reference category) on organizational identification. PF*Group - > Job satisfaction: Represents the interaction effect between procedural fairness EC and group membership on job satisfaction. PF*Group - > Identification - > JS: Represents the moderated mediation pathway from the procedural fairness EC × group membership interaction term (through organizational identification) to job satisfaction. Bolded term represents significant interaction effect.

In Samples 1a and 1b the interactions between EC procedural fairness and group membership on organizational identification and job satisfaction did not reach significance (all *p*s > 0.362), nor did the hypothesized moderated mediation pathways from this interaction term to job satisfaction (i.e., through organizational identification; all *p*s > 0.350). Conversely, in Sample 1c, the interaction between procedural fairness (LP) and group membership on organizational identification reached significance (*b* = −0.17, *SE* = 0.076, *p* = 0.048, 95% CI = [−0.328, −0.002]), and the hypothesized moderated mediation pathway approached statistical significance (*b* = −0.10, *SE* = 0.047, *p* = 0.053, 95% CI = [−0.196, 0.002]). A further investigation of these interactions revealed that, whereas the indirect effect of EC procedural fairness on job satisfaction was strong and significant among the minority workforce (*b* = 0.85, *SE* = 0.220, *p* < 0.001, 95% CI = [0.399, 1.294]), this effect was substantially smaller among the majority workforce—albeit still positive and significant (*b* = 0.11, *SE* = 0.45, *p* = 0.016, 95% CI = [0.020, 0.195]).

### 3.4 Discussion

Study 1 provided supportive evidence to our first two hypotheses. Specifically, it was shown that among minority group employees, procedural fairness perceptions concerning EC decision-making were consistently related to job satisfaction (*Hypothesis 1*), and that organizational identification was an explanatory process variable for this relationship (*Hypothesis 2*). Interestingly, the RW analyses on Samples 1b and 1c also demonstrated that our findings could not be attributed to a general and EC-specific procedural fairness confound. Lastly, we also obtained evidence in favor of Hypothesis 3b. That is, we found similar procedural fairness EC effects for majority and minority employees—although, in Sample 1c, this relationship was found to be less pronounced among majority members.

The main aim of Study 2 was to shed light on directionality. Specifically, although the Study 1 results provided converging evidence about the mediating role of organizational identification in the procedural fairness EC-job satisfaction relationship, it should nonetheless be acknowledged that mediation is, at its heart, a causal process, and causal inferences from cross-sectional data alone can be biased (Bullock et al., 2008). Hence, we conducted an additional longitudinal field study wherein we measured our focal variables at two different time points. If then, indeed, changes in the procedural fairness EC-job satisfaction relationship were found to be mediated by changes in organizational identification, this would provide strong additional evidence for the directionality envisaged in our hypothesized mediation model (and thus for *Hypothesis 2*). Furthermore, by sampling workers pertaining to different companies, we were able to control for various other workplace characteristics (e.g., company size, industry sector) and leadership work experiences (e.g., management experience, decision-making responsibilities at work), which could reasonably be expected to impact upon participants’ EC procedural fairness perceptions.

## 4 Study 2

### 4.1 Participants

Study 2 was preregistered at https://aspredicted.org/SBF_XRF. We recruited *N* = 313 Prolific workers at T1 (see our preregistration for a detailed summary of our sample size calculations). Given that the main aim of the present study was to provide an additional test of *Hypothesis 2*, we decided to limit our focus to the minority workforce. We excluded *n* = 20 because they either failed our attention check (“Please select the third response for this question”), or because they did not meet all of our inclusion criteria (i.e., (1) being a minority group member in their country of residence, (2) professing Islamic faith, and (3) being employed at the time of testing), resulting in a final T1 sample of *N* = 293 Muslim minority group members^8^ (150 males/142 females/1 non-binary; age: *M* = 30.94, *SD* = 8.39, range = 18–60).

About 3 months later, these participants were invited to complete our T2 survey. *N* = 285 (97.3% of the T1 sample) responded to our call. Exclusion of *n* = 20 who failed our attention check resulted in a final T2 sample of *N* = 265 (116 males; age: *M* = 29.45, *SD* = 8.18, range = 18–58; see Supplementary Tables A1, A2 for further demographic and work characteristics).

### 4.2 Measures

At T1, participants provided a few demographic and workplace characteristics and they completed the focal measures. At T2, all focal constructs were measured again using the same T1 scales. Unless stated otherwise, all measures were scaled 1 = completely disagree to 5 = completely agree. The full nine-item item Naumann and Bennett (2000) measure was administered to quantify EC procedural fairness perceptions (T1: *α* = 0.84; *M* = 3.55, *SD* = 0.75; T2: *α* = 0.89; *M* = 3.42, *SD* = 0.84). As before, these items were preceded by an introductory sentence and a few examples of EC issues. Furthermore, we also administered the full 14-item Leach et al. (2008) scale to measure organizational identification (T1: *α* = 0.85; *M* = 3.33, *SD* = 0.72; T2: *α* = 0.94; *M* = 3.32, *SD* = 0.84). General procedural fairness perceptions were measured with four items adapted from Liao and Rupp (2005). A sample item is “My organization’s procedures and guidelines are very fair.” The reliability of this scale was high (T1: *α* = 0.80; *M* = 3.95, *SD* = 0.81; T2: *α* = 0.86; *M* = 3.94, *SD* = 0.81). Job satisfaction was measured with the Study 1 single item (scaled 1 = very dissatisfied to 5 = very dissatisfied, T1: *M* = 3.08*, SD* = 1.43; T2: *M* = 3.62, *SD* = 1.09).

### 4.3 Data-analysis and results

#### 4.3.1 Correlation analysis

Supplementary Table B4 displays the T1 and T2 correlation matrices. As can be derived from this table, EC procedural fairness and job satisfaction were positively and significantly associated across both measurement occasions (*r*s = 0.22–0.45, both *p*s < 0.001). These findings thus provide clear evidence for *Hypothesis 1*.

#### 4.3.2 EC vs. general procedural fairness: confirmatory factor analyses

As in Study 1, we first verified whether EC and general procedural fairness perceptions could indeed empirically be distinguished. The results of our CFAs are reported in detail in the Section C, Supplementary Tables C3, C4. It was shown that the two-factor model significantly outperformed the model with a single, latent fairness factor (T1: *χ^2^*(50) = 109.94, *p* < 0.001, *χ^2^/df* = 2.20, CFI = 0.96, TLI = 0.94, RMSEA = 0.06 [0.05, 0.08], SRMR = 0.05; LRT = *χ^2^* (1) = 400.40, *p* < 0.001; T2: *χ^2^*(47) = 85.64, *p* < 0.001, *χ^2^/df* = 1.82, CFI = 0.98, TLI = 0.97, RMSEA = 0.05 [0.03, 0.07], SRMR = 0.06; LRT = *χ^2^* (1) = 516.50, *p* < 0.001).

#### 4.3.3 EC vs. general procedural fairness: relative weight analyses

Analogous to Study 1, we assessed the unique influence of EC and general procedural fairness perceptions on the dependent variable. The results of our RW analyses revealed that EC and general procedural fairness were each uniquely associated with job satisfaction (T1: EC: *b* = 0.02, 95% CI = [0.007, 0.059]; general: *b* = 0.13, 95% CI = [0.061, 0.209]; T2: EC: *b* = 0.11, 95% CI = [0.056, 0.166]; general: *b* = 0.26, 95% CI = [0.182, 0.343]), as such additionally corroborating *Hypothesis 1* and our contention that EC and general, non-EC fairness perceptions can empirically be distinguished in organizational settings.

#### 4.3.4 Cross-sectional mediation analysis

In support of *Hypothesis 2*, our analysis revealed that the relationship between EC procedural fairness and job satisfaction was mediated by organizational identification, both at T1 (*b* = 0.49, *SE* = 0.063, *p* < 0.001, 95% CI = [0.362, 0.610]) and T2 (*b* = 0.54, *SE* = 0.061, *p* < 0.001, 95% CI = [0.422, 0.661]).

#### 4.3.5 Longitudinal mediation analysis

**Figure 3:**
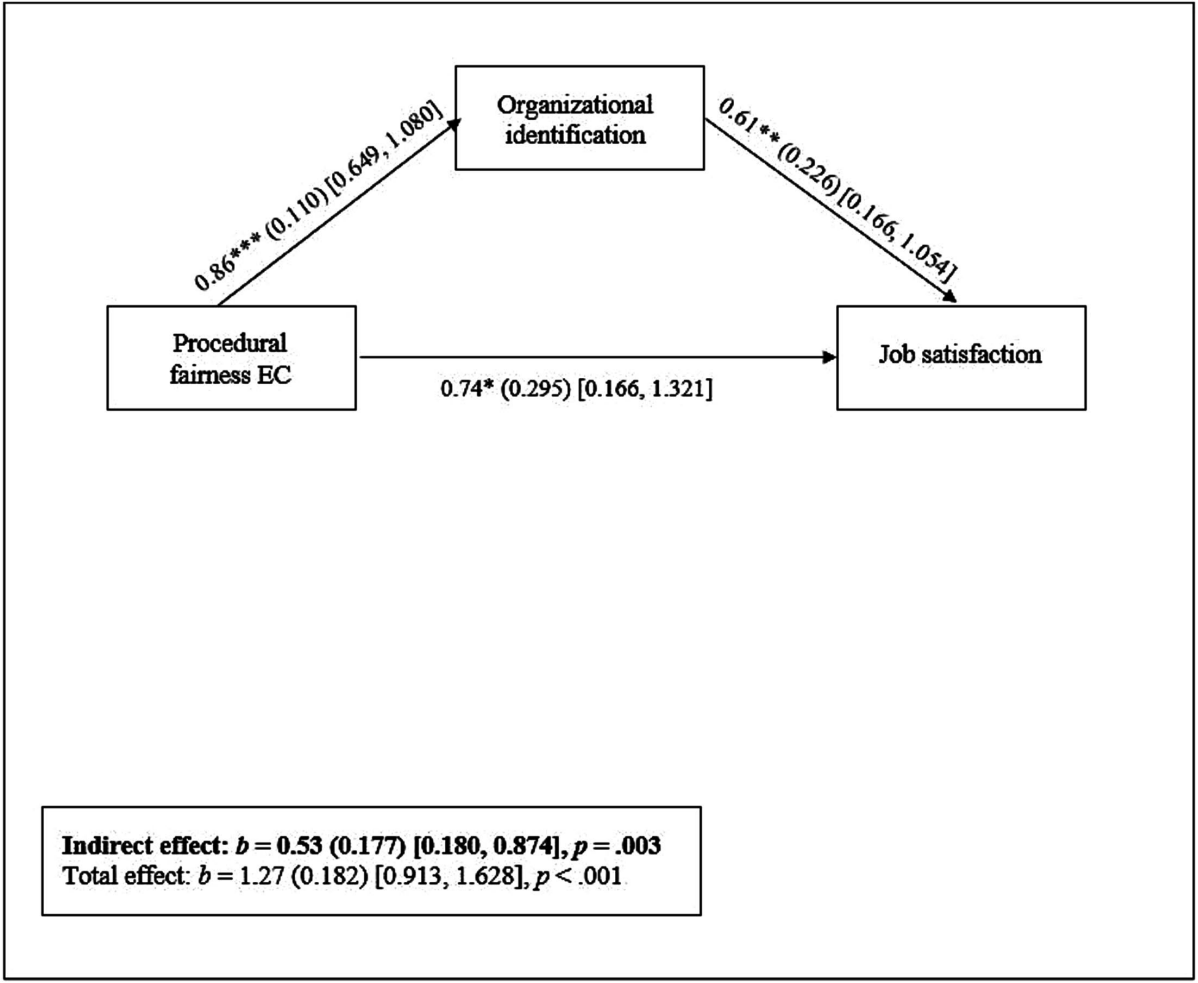
Schematic overview of results of multilevel mediation analysis (Study 2) with EC procedural fairness as predictor, organizational identification as mediator, and job satisfaction as dependent variable. Notes. ** *p* < 0.05, * *p* < 0.01, *** *p* < 0.001. Statistically controlled for age, gender, education level, income, organizational tenure, percentage of union members working in organization, employment status, type of work (blue vs white collar), work hours per week, company size, number of subordinates, supervisory and decision-making responsibilities, and management experience. Reported effects are unstandardized [standard errors in parentheses, and 95% confidence intervals between square brackets].

To assess whether changes in the EC procedural fairness-job satisfaction relationship were mediated by changes in organizational identification—and thus, to answer Study 2’s focal research question—we fitted a multilevel mediation model with the Lavaan package in R. In this model, repeated measurement occasions were entered as the level-1 variable, and participants as the level-2 cluster variable.^9^^,^^10^ See Online Appendix (Section D) for a detailed overview of our mixed-model procedure. Supporting *Hypothesis 2*, the longitudinal relationship between EC procedural fairness and job satisfaction was indeed mediated by organizational identification (*b* = 0.53, *SE* = 0.177, *p* = 0.003, 95% CI = [0.180, 0.874]). Figure 3 provides an overview of the estimated model.^11^

### 4.4 Discussion

Study 2 provided further support for our hypotheses. First, it was shown that among Muslim minority group employees working in various companies, countries and industries, EC procedural fairness perceptions were consistently associated with job satisfaction, both on the individual measurement occasions and longitudinally. Moreover, and in line with the Study 1 findings, our RW analyses demonstrated that EC procedural fairness perceptions explained variance in job satisfaction, above and beyond general procedural fairness perceptions. Taken together, these findings strongly supported *Hypothesis 1*.

Most importantly, our multilevel mediation analyses further revealed that organizational identification was an explanatory process variable for the longitudinal relationship between EC procedural fairness and job satisfaction (*Hypothesis 2*). The latter results align with *Hypothesis 2*, and they thereby deliver additional support for the causal chain encapsulated in our hypothesized mediation model.

Next, we conducted Study 3 to further scrutinize the present effects. Specifically, notwithstanding that we rigorously controlled for a variety of worker and work environmental characteristics in the Study 1 and 2 samples, we also recognize that employees do not operate in a social vacuum. In fact, contemporary organizational settings are often organized in smaller collaborative work teams. As an example, it has been shown that, over the last 20 years, workplace collaboration has increased by at least 50%, and more than 50% of workers in the United States report that their jobs are reliant on collaboration (Boskamp, 2022). It stands to reason that the processes and dynamics operating within such collaborative teams further shape employees’ job satisfaction, and not taking these within-group processes and dynamics into account may hamper the accurate estimation of the “pure” effect of EC procedural fairness perceptions. In this regard, it should be noted that multilevel modeling—whereby individuals (e.g., team members) are studied in “nested” units (e.g., teams)—is a powerful approach, which circumvents some of the obstacles raised by standard analyses (Muthén and Asparouhov, 2011). For example, it has been argued that “multilevel theories can help illuminate the context surrounding individual-level processes, clarifying precisely when and where such processes are likely to occur within organizations” (Klein et al., 1999). More importantly, multilevel models also allow to statistically control for some micro-level workplace-specific attributes, thereby providing a more accurate estimation of the processes at play at the individual level. Hence, in Study 3, to rigorously control for work environmental characteristics and within-team dynamics, we collected a large sample of employees pertaining to various work teams *within* the same company and analyzed their data using multilevel modeling.

## 5 Study 3

### 5.1 Participants

*N* = 509 assembly line workers completed either a paper-and-pencil or online survey (211 minority group members;^12^ 108 teams; median age range = <30 years). Team leaders provided each of their subordinates with a paper copy of our questionnaire and an envelope which could be sealed to return the responses anonymously. For the web-based surveys, team leaders provided their subordinates with a weblink and/or a QR code. A power sensitivity analysis further revealed that our study had 80% power to detect a minimal slope for our mediator of size *b* = 0.15.

### 5.2 Measures

At T1, *EC procedural fairness* was measured with four items by van den Bos et al. (2001) (*α* = 0.91; *M* = 3.31, *SD* = 0.83). This question was preceded by the same introductory sentence as in Study 1. Sample items are “We are treated fairly when decisions are made that concern such cultural, ethnic and linguistic issues” and “We are able to voice our opinions when decisions are made that concern such cultural, ethnic and linguistic issues.” *Organizational identification* (*α* = 0.88; *M* = 3.60, *SD* = 0.88) and *job satisfaction* (*M* = 3.69, *SD* = 0.96) were measured with the same items as in Study 1. We further administered one item gauging perceived team leader *general procedural fairness*, i.e., “In what way are decisions generally made by your team supervisor? We are treated fairly” (scaled 1 = completely disagree, 5 = completely agree; *M* = 3.29, *SD* = 0.95).

### 5.3 Data-analysis and results

#### 5.3.1 Correlation analysis

Supplementary Table B5 displays the correlations among our focal variables. In line with *Hypothesis 1*, EC procedural fairness and job satisfaction were positively and significantly associated (*r* = 0.30, *p* < 0.001).

#### 5.3.2 EC vs. general procedural fairness: confirmatory factor analyses

As in Studies 1 and 2, we first verified whether EC and general procedural fairness perceptions could indeed empirically be distinguished. The results of our CFA are reported in detail in the Section C, Supplementary Table C5. Like before, it was shown that the two-factor model significantly outperformed the model with a single, latent fairness factor (*χ^2^*(4) = 16.73, *p* = 0.002, *χ^2^/df* = 4.18, CFI = 0.99, TLI = 0.98, RMSEA = 0.08 [0.05, 0.13], SRMR = 0.02; LRT = *χ^2^* (1) = 64.61, *p* < 0.001).

#### 5.3.3 EC vs. general procedural fairness: relative weight analyses

EC and team leader procedural fairness were each uniquely associated with both organizational identification (EC: *b* = 0.07, 95% CI = [0.034, 0.133], *p* < 0.05; team leader: *b* = 0.16, 95% CI = [0.093, 0.228], *p* < 0.05) and job satisfaction (EC: *b* = 0.07, 95% CI = [0.030, 0.126], *p* < 0.05; team leader: *b* = 0.17, 95% CI = [0.107, 0.248], *p* < 0.05), as such providing further evidence for *Hypothesis 1* and our contention that EC and general, non-EC fairness perceptions can empirically be distinguished in organizational settings.

#### 5.3.4 Multilevel mediation and moderated mediation analysis

**Figure 4:**
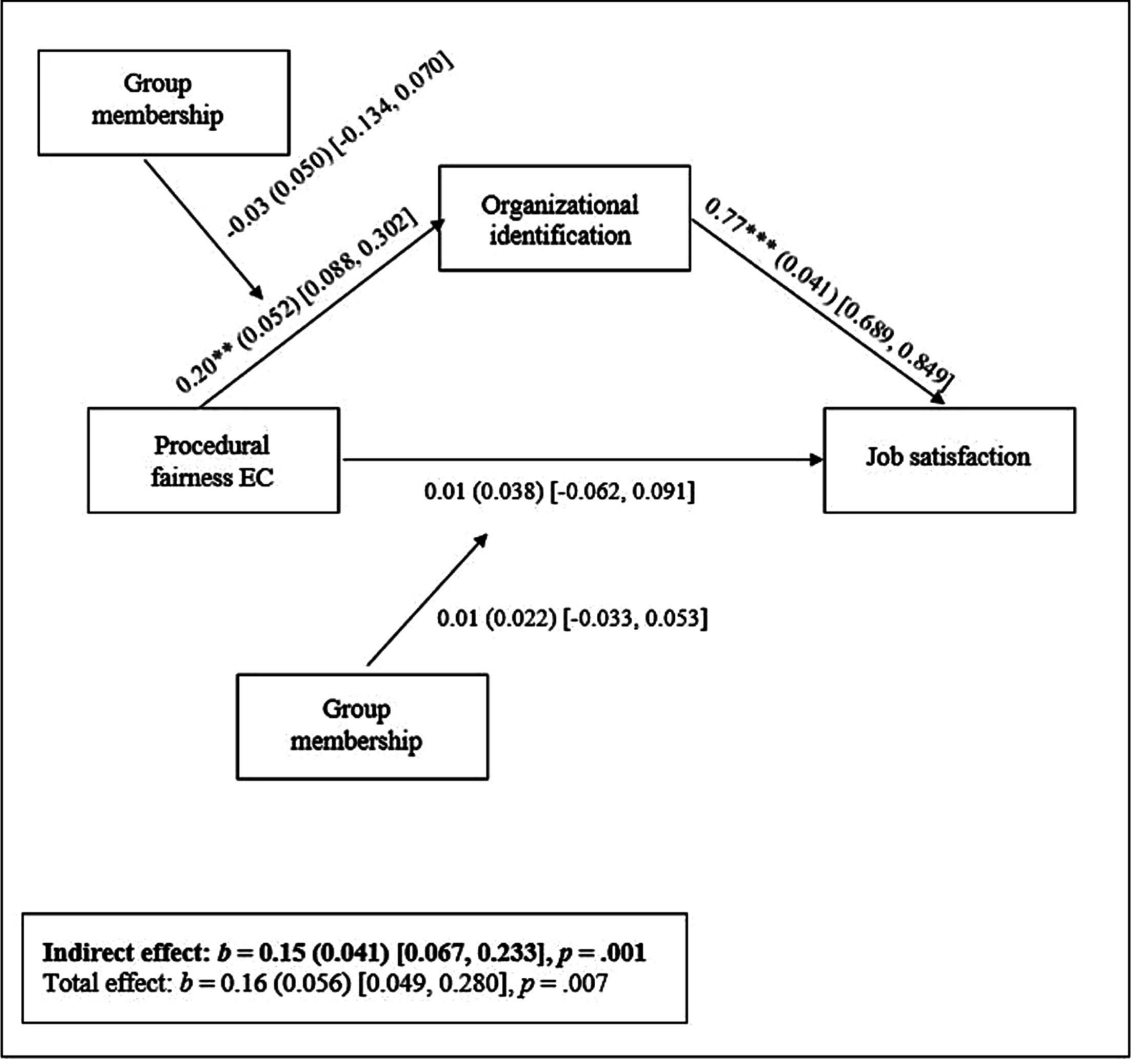
Schematic overview of results of multilevel moderated mediation analysis (Study 2) with EC procedural fairness as predictor, organizational identification as mediator, group membership (1 = majority group, else 0) as moderator, and job satisfaction as dependent variable. Notes. ** *p* < 0.01, *** *p* < 0.001. Statistically controlled for age, education level, type of contract and team tenure. Reported effects are unstandardized [standard errors in parentheses, and 95% confidence intervals between square brackets].

We then fitted a multilevel (moderated) mediation model for the entire sample with participants as level-1 variable and team as level-2 clustering variable, while controlling for our covariates.^13^^,^^14^ See Online Appendix (Section D) for a detailed overview of our mixed-model procedure. Supporting *Hypothesis 2*, results of our analysis revealed that the relationship between EC procedural fairness and job satisfaction was mediated by organizational identification (*b* = 0.15, *SE* = 0.041, *p* = 0.001, 95% CI = [0.067, 0.233]). Furthermore, none of the interactions between procedural fairness and group membership reached significance (all *b*s < |0.19|, all *p*s > 0.521), a finding which is in line with the predictions made in *Hypothesis 3b*. Figure 4 provides an overview of the estimated model.

### 5.4 Discussion

The results of Study 3 replicated and extended those of Study 1 (and Study 2). Firstly, our multilevel analyses provided additional evidence for the robustness of our mediation model (*Hypotheses 1 and 2*). Secondly, in line with *Hypothesis 3b*, no significant EC procedural fairness × group membership interactions were obtained.

## 6 Internal meta-analysis

To further assess the robustness of our findings, we conducted an internal meta-analysis. We accounted for the substantial between-sample heterogeneity (in terms of employee, labor and workplace characteristics) by adding random, study-specific effects to the statistical model used to calculate the meta-analytic effect (Hedges and Olkin, 1985). Moreover, given the small number of samples (i.e., *n* = 5), we decided to conduct a Bayesian random-effects meta-analysis with the *bayesmeta* R package (Röver, 2020). In this analysis, a posterior distribution was computed per effect size, and for the heterogeneity parameter *τ*—which models between-study variation.

For our meta-analysis, we focused on the strength of the indirect relationship between EC procedural fairness and job satisfaction (for minority and majority separately; and for the full samples), and the moderating effect of group membership. To this end, we first computed study-specific standardized effect sizes (*β*s) which were then transformed into partial correlation coefficients for further processing (see Röver, 2020). An informative normal prior (*μ* = 0, *σ* = 4) was used to infer the posterior distribution of the effect sizes, and a proper informative half-normal prior (scaling parameter 0.5) was used to approximate the prior distribution of the heterogeneity parameters τ. Table 2 presents the point estimates of the posterior means of all effect sizes, along with their 95% credibility intervals [Cis] and τs.

**Table 2:** Results of internal meta-analysis: point estimates of the means of posterior distributions of true meta-analytic effect sizes, 95% credibility intervals, and corresponding heterogeneity parameters *τ*.

Effect	Effect size	95% credibility interval		*τ*
		*LL*	*UL*	
PF - > JS Minority	0.35	0.14	0.57	0.15
PF - > JS Majority	0.18	0.01	0.35	0.13
PF - > JS Overall	0.19	0.07	0.32	0.08
PF*Group Mod. med.	−0.03	−0.16	0.10	0.09

All effect sizes represent partial correlation coefficients. PF - > JS Minority: indirect effect of EC procedural fairness on job satisfaction (through organizational identification) among minority group employees. PF - > JS Majority: indirect effect of EC procedural fairness on job satisfaction (through organizational identification) among majority group employees. PF - > JS Overall: indirect effect of EC procedural fairness on job satisfaction (through organizational identification) in the full sample. PF*Group Mod. med.: moderated mediation effect of EC procedural fairness * group membership on job satisfaction (through organizational identification).

Corroborating *Hypothesis 1 and 2*, the results revealed that the pooled mediation effect size among minority group employees was *B* = 0.35 (*SD* = 0.105; 95% Ci = [0.144, 0.567]), indicating a moderate overall indirect effect of EC procedural fairness on job satisfaction in this population. Furthermore, the pooled mediation effect size among majority group employees was 0.18 (*SD* = 0.086; 95% Ci = [0.008, 0.351]), indicating a moderate overall indirect effect of EC procedural fairness among this population. It can thus be concluded that the EC procedural fairness effects we encountered were all very likely to be practically meaningful. Conversely, the pooled effect size for the EC procedural fairness × group membership pathway was −0.03 (*SD* = 0.067; 95% Ci = [−0.162, 0.099]), indicating insufficient evidence for moderated mediation, thus supporting *Hypothesis 3b*.

## 7 General discussion

The present research investigated the relationship between the perceived procedural fairness of EC decision-making and job satisfaction. Integrating Guillaume et al.’s (2014) model of diversity management with the CPF model (Dierckx et al., 2024; Valcke et al., 2020a, 2020b), we hypothesized that minority employees’ procedural fairness perceptions with respect to EC decisions are positively related to their organizational identification, and hence, their job satisfaction. Moreover, we also examined the reactions of the majority group workforce.

### 7.1 Main findings

The results of all three studies provided converging evidence for these predictions. In Study 1, in agreement of *Hypothesis 1*, EC procedural fairness perceptions were positively associated with job satisfaction among minority group members. Moreover, as predicted by *Hypothesis 2*, organizational identification fully mediated this relationship. Additionally, in all three Study 1 samples, we found very similar EC procedural fairness effects for majority and minority group employees, a finding which corroborates *Hypothesis 3b*.

The robustness of the Study 1 results was further substantiated in two follow-up studies, as such corroborating *Hypotheses 1*, *2* and *3b*. That is, Study 2 employed a longitudinal design and provided further evidence for the causal sequence in our hypothesized mediation model. In addition, Study 3 replicated the Study 1 findings using multilevel techniques in a sample of blue-collar workers pertaining to >100 ethnically diverse work teams in one organization. Moreover, the results of an internal meta-analysis further corroborated our hypotheses and thereby additionally attested to the credibility of the presented study-specific results. Overall, the present results thus converge and highlight the critical potential of EC procedural fairness for organizational diversity management.

### 7.2 Theoretical contributions

Our findings contribute to the field of organizational psychology in various ways. First and foremost, they show the relevance of procedural fairness in EC decision-making, and, as such, for managing organizational EC diversity. In their diversity management model, Guillaume et al. (2014) have emphasized the critical importance of diversity policies and procedures that promote an inclusive work environment, which can foster minority group members to “feel at home” and thrive in the workplace. As a few examples, these scholars highlighted coaching and mentoring of underrepresented demographic groups, equal mobility opportunities, and equal pay as promising avenues to bolster an inclusive work environment. Most of these policies concern “distributive” issues as they involve tangible outcomes. Nonetheless, these authors also encouraged researchers to further explore “other *policies* and *procedures* that evoke favorable climates for inclusion in organizations or work groups” (p. 796). In heeding this call, the present research demonstrates that decision-makers—team leaders and managerial supervisors—can accommodate their EC minority employees, and therefore contribute to effective organizational EC diversity management, by applying the principles of procedural fairness to issues that exclusively concern the EC minority workforce, that is, to EC issues.

A further noteworthy aspect of the present findings is that these EC procedural fairness effects emerged, even though the relationship between the fairness provider and the recipient is not as straightforward as in the standard or “classical” fairness situation. That is, the bulk of procedural fairness studies have focused on decisions whereby individual employees (e.g., Colquitt et al., 2023; Konovsky, 2000; Van den Bos, 2001) or work units (Li and Cropanzano, 2009; Liao and Rupp, 2005; Naumann and Bennett, 2000; Raineri, 2023) are directly targeted. Conversely, in EC decision-making, the relationship between the provider and the recipient of the (un)fair treatment is often more subtle. That is, decisions involving ethnic, cultural, and linguistic issues often do not necessarily directly involve individual minority group members. In fact, sometimes other minority employees can even be unaffected by the outcome. Despite this critical nuance, our results have shown that EC procedural fairness perceptions impact upon organizational identification and job satisfaction to a similar extent as theirs general counterpart does.

Thirdly, the present findings corroborate our hypothesized theoretical model, because it was shown that organizational identification consistently mediated the EC procedural fairness-job satisfaction association. In this regard, they align with Guillaume et al. (2014) conjecture that identity concern satisfaction lies at the root of effective diversity management procedures. On a broader level, the latter results also converge with the CPF model, which contends that EC procedural fairness effects occur because EC fair treatment induces a temporary redefinition of oneself as an EC minority group member, which in turn elicits more active forms of identification with the superordinate category embodied by the specific authority figure (Valcke et al., 2020b). Given that the relevant decision-makers in the current study (e.g., team supervisors, management) were all representatives of their organizations at large, the currently observed mediation effect of organizational identification can thus be interpreted as a prototypical demonstration of how EC procedural fairness perceptions foster “superordinate category identification”—as predicted by the CPF model. Moreover, a fourth theoretical contribution of our work is that it also allows for a direct comparison of minority and majority responses to fair treatment that specifically targets minority employees. Our results consistently revealed that EC procedural fairness perceptions were similarly positively related to organizational identification and job satisfaction among the majority workforce. In this vein, the present set of findings seems to align with our conjecture in the Introduction that EC procedural fairness enactment can promote intergroup liaisons: To the extent that minority employees interpret equally enthusiastic responses among majority group members as a sign of allyship and involvement, EC procedural fairness may undoubtedly contribute to bridging the divide between the minority and majority workforce.

At first sight, these results thus provide strong support for our deontic model-based predictions (Folger, 2001), and no support for our conflict account (rooted in Sherif, 1966). Nonetheless, it should be acknowledged that we did not explicitly gage participants’ underlying motives (as this fell out of the scope of the current research), and it is therefore difficult to assess which specific moral standards shape majority group members’ responses to EC procedural fairness. According to deontic theory, most people value fairness for its own sake (e.g., Turillo et al., 2002), and they believe that decision-makers have the universal moral obligation to grant *every* person the fair treatment he/she deserves (Barclay et al., 2017; Beugré, 2012). Although this explanation aligns well with the present results, we can nonetheless envisage alternative moral motivations for the observed behavior. For example, research has demonstrated that, on average, majority group members believe that their dominant group status comes with enhanced moral responsibilities toward members of disadvantaged groups (Dierckx et al., 2022). Consequently, they weigh their own behavior and that of their majority peers in terms of enhanced moral obligations, and they feel that minority group members are particularly entitled to receive rightful and unbiased treatment from the majority group. It stands to reason that evaluating oneself and one’s peers in terms of such *specific* moral standards (i.e., directed toward minority group members in particular) would undoubtedly result in similar behavior to that observed in our studies. At any rate, we caution against overly confident interpretation of our results in terms of *universal* or *deontic* moral obligations (i.e., toward “all humanity”).

### 7.3 Practical recommendations on diversity management

Besides these theoretical considerations, the present results also have some important managerial implications. As we argued in the Introduction, organizational decision-makers will increasingly have to operate as company “diversity managers” (Homan et al., 2020) and resolve a mounting number of EC issues. That is, company managers will increasingly need to delineate the extent to which they can accommodate religious practices in the workplace, improve the visibility of the ethnic minority workforce in leadership positions, incorporate dietary preferences of minority groups into their company cafeteria menu, and develop leave regulations that take religious and secular holidays into account, and so on. The results of our three studies converge in this regard and highlight that, by striving to achieve that decision procedures vis-à-vis such matters are perceived as fair and unbiased, managers can straightforwardly contribute to the organizational identification and job satisfaction of their workforce.

How, then, can organizational decision-makers increase employees’ perceptions of justice? A first potential avenue is that managers assign individual minority employees to operate as “representatives” of their respective minority groups and involve them in in the resolution of EC issues and the development of diversity policies. Not only could these representatives provide the management with “bottom-up” input from their peers and as such enhance the minority influence in the conception of organizational policies, they could further also be tasked with the “top-down” communication of relevant managerial decisions and strategies.

Another way of granting minority groups “voice” in EC decision-making—and, as such, enhance their EC procedural fairness perceptions—is to organize weekly or monthly meetings wherein the entire EC workforce can, on a voluntary basis, engage in face-to-face communication with the company diversity managers. These meetings could constitute an opportunity for management to communicate clearly, accurately and openly about their EC decisions, the reasons behind them, and the way they were formed. Additionally, these meetings could allow minority employees to feed back to the management about how they have experienced their decisions and policies, in order to help them gain greater understanding of the consequences and inspire them to make any necessary modifications to their decisions, if needed.

Third, given that minority group members tend to attach critical importance to bias suppression and carefully screen organizational procedures for evidence of bias/lack of bias displayed by the decision-maker (Dierckx et al., 2020b), another promising avenue to promote perceptions of EC procedural justice may be to “source out” certain diversity management decisions to specialized HR agencies or university research units. By relying on outsiders, organizations can assure their minority workforce that the decision-maker has no personal interest in the allocation decision—which ultimately could result in enhanced perceptions of bias suppression.

### 7.4 Strengths, limitations, and directions of future research

Taken together, our research endeavor has provided converging cross-sectional, longitudinal, multilevel, and meta-analytical evidence for our core hypotheses. Of course, the present studies also suffer from some limitations. First, it should be noted that, except for Study 2, our samples consisted mainly of blue-collar workers (e.g., professional cleaners in Study 1, Sample 1c, or assembly line workers in Study 3). In this regard, our research clearly diverges from the bulk of fairness research, which has largely investigated predominantly white-collar samples (e.g., Eib et al., 2021; Sweeney and McFarlin, 1993). Although the present research thus additionally contributes to literature by focusing on this underrepresented subgroup of the organizational workforce, it nonetheless remains to be shown whether the present results hold among white-collar samples.

Second, although Study 1 and Study 3 consistently showed that majority group membership does not *directly* moderate the relationship between EC procedural fairness and job satisfaction, we cannot exclude the possibility that some specific clusters of people within the majority group may react otherwise. For example, it has been shown that those who strongly endorse right-wing ideological attitudes are more likely to oppose diversity policies (Pratto et al., 1994). Taking this into account, we can envisage that the relationship between EC procedural fairness and job satisfaction becomes less pronounced, or even negative, among right-wing adherent majority members. These are, of course, questions that future research should address.

Third, the current results were obtained in the European cultural context, i.e., Belgium (Studies 1 and 3) and among several European countries (Study 2), and it therefore remains to be shown that the observed reactions to EC procedural fairness would generalize to regions with different social dynamics. Nonetheless, a recent study (Dierckx et al., 2024) demonstrated similar—*albeit* somewhat weaker—EC procedural fairness effects in the South African context. Given that Africa and Europe differ on a number of socio-economic dimensions, the latter study thus suggests some degree of universality of the EC procedural fairness effects observed in the current study.

Fourth, it should be noted that our samples differed significantly in terms of context (e.g., national vs. cross-national) and background characteristics (e.g., industrial sector, diversity, gender balance, etc.). On the one hand, it may be contended that this substantial heterogeneity raises questions about the generalizability of our study-specific findings. For instance, in Study 1 (Sample 1c) the procedural fairness-job satisfaction relationship was found to be somewhat weaker among the majority group workforce. Given that this sample consisted exclusively of female employees, one might consider these results indicative of a gender imbalance in EC procedural fairness effects. On the other hand, we must stress that Studies 2 and 3 largely alleviate such concerns, as both these studies showed that the basic findings from Study 1 held while controlling for a plethora of employee background (Study 2) and work environment (Study 3) characteristics. At any rate, future studies using large, well-powered samples from all across the world are needed to further substantiate the present model’s generalizability.

Finally, we hasten to emphasize that the diversity management model of Guillaume et al. (2014) was taken as a theoretical background from which we derived our specific research questions. We however by no means wish to claim that we extended our further developed this model. That being said, we do believe that the current set of findings can inspire future research that directly investigates the link between our work and that of Guillaume et al. (2014). For example, follow-up studies may examine whether the procedurally fair resolution of EC issues does indeed directly foster perceptions of an “inclusive work environment.” Relatedly, researchers may want explore whether this relationship is mediated and/or conditioned by the extent to which minority employees’ identity concerns (e.g., their need for belongingness) are addressed. In sum, we strongly encourage future research endeavors that go beyond the present contribution and meaningfully extend of the work of Guillaume et al. (2014).

### 7.5 Concluding remarks

The present research examined the relationship between procedural fairness perceptions with respect to ethnic-cultural issues and job satisfaction in the workforce of various companies. Despite a few noteworthy studies demonstrating the importance of resolving EC issues in non-organizational settings in a procedurally fair way, our study was the first to explore EC procedural fairness effects within the organizational cosmos. In doing so, we employed a dual focus, examining both minority and majority group members’ perceptions. Remarkably similar results were obtained across five samples which varied considerably in terms of employee characteristics, industrial sector, and the source of ethnic-cultural procedural fairness (i.e., team leader, management, etc.). As expected, minority employees’ ethnic-cultural fairness perceptions were positively related to organizational identification, and subsequently, their job satisfaction. Interestingly, similar results were obtained for majority employees as well—although this group is not the prime beneficiary of such decisions. Taken together, at the theoretical level, our results call for enhanced attention to the psychological processes, elicited by procedural fairness, that operate at the *collective* levels of the social self. At the same time, at the practical level, our findings underscore the importance of studying the concrete implementation of procedural fairness in ethnic-cultural issues arising in the workplace to effectively manage the diverse 21st century workforce.

## Data Availability

The datasets presented in this study can be found in online repositories. The names of the repository/repositories and accession number(s) can be found in the article/Supplementary material.

## Footnotes

^1^Throughout the manuscript, we will refer to evaluations of decision-making processes without an ethnic-cultural component as *general* procedural fairness perceptions, to distinguish them from EC (procedural fairness) perceptions.

^2^Note that, according to Guillaume et al.’s (2014) model, inclusion-promoting interventions can also increase minority group members’ self-efficacy beliefs, which could, in turn, enhance their job satisfaction. Empirical scrutinization of the latter contention fell, however, out of the scope of the current study.

^3^In Study 1, participants were considered EC minority group employees when they themselves were born abroad, and/or at least one of their parents or grandparents was born abroad. This definition is commonly used in ethnic minority research to distinguish between those belonging to minority groups and those belonging to the national majority (e.g., Brans et al., 2004; Valcke et al., 2022).

^4^In the Sample 1c questionnaire, we gauged perceptions vis-à-vis a specific EC decision. Hence, this last sentence was replaced by “In [Company] the management asks you to speak Dutch at work. The following statements refer to this language policy.”

^5^The item “In the course of the decision procedure, [company] does not show any preference for a specific group of employees” was dropped, because it could be interpreted as referring to one’s ethnic, cultural or linguistic group, and thus to EC decision making.

^6^In Sample 1b, a third item was added to measure this variable “Being an employee of [company] is an important part of my identity.”

^7^For an overview of all covariate analyses and a summary of which covariates were added to the mediation models, see document “Covariate analyses” on our Open Science webpage.

^8^In Study 2, participants were considered ethnic-cultural minority group employees when they indicated that they professed Islamic faith, and when they reported to belong to another ethnic-cultural group than the numerical majority in their respective country (e.g., they were non-White citizens living and working in the UK).

^9^We acknowledge that participants were also nested within countries. As such, we first attempted to fit a multilevel mediation model with repeated measurement occasions as level-1 variable, participants as level-2 cluster variable, and country as level-3 cluster variable. This model, however, did not converge, due to the small number of observations in some countries.

^10^For an overview of all covariate analyses and a summary of which covariates were added to the mediation models, see document “Covariate analyses” on our Open Science webpage.

^11^As an additional analysis, we computed T2-T1 difference scores for all three focal variables and fitted a mediation model with (T2-T1) EC procedural fairness as predictor, (T2-T1) organizational identification as mediator, and (T2-T1) job satisfaction as dependent variable. Analogous to our multilevel modeling output, the results revealed that the longitudinal relationship between EC procedural fairness and job satisfaction was mediated by organizational identification (b = 0.40, SE = 0.066, *p* < 0.001, 95% CI = [0.269, 0.527]).

^12^Our minority employee group inclusion criteria were analogous to Study 1.

^13^Analogous to Study 1, we first attempted to fit the simple multilevel mediation model for the minority group subsample. This model, however, did not converge, due to the small number of observations per team cluster (max *n* = 9, M = 2.71).

^14^For an overview of all covariate analyses and a summary of which covariates were added to the mediation models, see document “Covariate analyses” on our Open Science webpage.
