# Supplementary material for: Ethnic-cultural procedural fairness effects on organizational identification and job satisfaction among minority and majority employees
Source: Front Psychol. 2025 Apr 15;16:1445469. doi: 10.3389/fpsyg.2025.1445469 (PMC12037562; doi:10.3389/fpsyg.2025.1445469)
Supplement: Supplementary file 1 [file Table_1.docx]

These are the supplementary materials for the covariate analyses of “ETHNIC-CULTURAL PROCEDURAL FAIRNESS EFFECTS ON ORGANIZATIONAL IDENTIFICATION AND JOB SATISFACTION AMONG MINORITY AND MAJORITY EMPLOYEES ”. Below, we first present all the sample-specific covariates and our rationale for the inclusion of these constructs. Then, we provide a detailed description of the analyses we conducted with respect to these covariates.

# **Overview of included covariates**

**Sample 1a, Study 1**

We included a variety of control variables. *Interactional fairness perceptions* (with respect to ethnic-cultural decision-making) were assessed with two items, based on Colquitt et al (2001), i.e., “We are treated with respect” and “We are adequately informed” (*α* = .72; *M* = 3.56, *SD* = 0.92). Furthermore, given that both *perceived diversity* and *diversity beliefs* have been (negatively) associated with identification and performance in organizations (e.g., Hentschel et al., 2013; Shemla et al., 2016; Van Knippenberg et al., 2007), we gauged these perceptions with two single-item measures: “How many members of your team belong to another ethnic-cultural group than yours?” (i.e., perceived diversity; rated on a scale ranging from 1 = none to 10 = all of them; *M*  = 3.54, *SD* = 2.25), and “Is diversity in the workplace something that you value?” (i.e., diversity beliefs; rated on a scale ranging from 1 = not at all, to 5 = very much; *M*  = 4.09, *SD* = 1.18). One item was also implemented to measure the *strength of* participants’ *religious affiliation*: “How important is religion for you personally?” (rated on a scale ranging from 1 = not important at all, to 5 = very important; *M*  = 2.62, *SD* = 1.18). Finally, we also recorded whether participants were *temporarily or permanently employed*, whether they had a *fulltime or part time contract*, whether they were a *member* of the *union*, which *shift* they were currently working (day, night or weekend), which *company site* they were working at and their *organizational tenure* (i.e., the number of years working in the organization).

**Sample 1b, Study 1**

We measured general (i.e., non-EC) procedural fairness perceptions with five out of the six items used to measure EC procedural fairness^[[1]](#footnote-1)^. However, unlike the former scale, the introductory sentences did not refer to “decisions… which affect an entire group of people belonging to the same ethnic-cultural group”, but rather to ”In general, how are decisions made in [company]?”. The reliability of this scale was acceptable (*α* = .77; *M* = 2.75, *SD* = 0.73).

Apart from general fairness, we also included the Sample 1a *perceived diversity* (*M* = 1.82, *SD* = 2.12), *diversity beliefs* (*M* = 3.19, *SD* = 1.11) and *strength of religious affiliation* items (*M* = 3.04, *SD* = 1.11). Three items were implemented to assess participants’ *perspectives on cultural diversity* (*α * = .71; *M* = 3.52, *SD* = 0.85): “The presence of foreign cultures enriches our society”, “People belonging to different cultural groups should minimize their interactions” (reverse-scored) and “There is not enough concern for people belonging to a different culture in Belgium”. Finally, we also recorded whether participants were a *member* of the *union*, which *company branch* they were working at, the *type of labor* (blue vs white collar) and their *organizational tenure*.

**Sample 1c, Study 1**

Like in Sample 1b, we controlled for general (i.e., non-EC) fairness perceptions with one item assessing one’s job coach’s decision-making (“When my job coach makes decisions that concern me, he/she does it in a fair way”; *M* = 4.17, *SD* = 0.80). Furthermore, given the specific issue, we also recorded participants’ *Dutch language proficiency* (1 = not proficient at all, 5 = very proficient; *M*  = 4.40*, SD* = 0.93).

**Study 2**

Like before, we controlled for *general* (i.e., non-EC) *fairness perceptions* with four items adapted from Liao and Rupp (2005). A sample item is “My organization’s procedures and guidelines are very fair”. The reliability of this scale was high (T1: *α* = .80; *M* = 3.95, *SD* = 0.81; T2: *α* = .86; *M* = 3.94, *SD* = 0.81). Employment status was assessed with one item: “What is your employment status?” (response options: full-time, part-time, unemployed, not in paid work (e.g., retired, disabled,...), other). Furthermore, participants were also asked to estimate the percentage of union workers in their organization (scaled 0-100%; *M* = 44.24, *SD* = 37.58).

To gauge the extent to which participants were in a subordinate position at work, we probed whether or not they had a *direct supervisor* at work. To gauge the extent to which participants were involved in leadership and decision-making processes at work, we probed whether they had *management experience*, whether they had any *supervisory responsibilities*, whether they had any *decision-making responsibilities* at work, and the *amount of people they had the authority to give instructions to*.

Finally, we also recorded which *shift* participants were currently working (day, night, rotating with or without night shifts, other)*,* the *type of labor* (blue vs white collar), the number of *weekly worked hours*,their *company’s size* (i.e., in terms of the number of people working there), and their *organizational tenure*.

**Study 3**

Like before, we controlled for general (i.e., non-EC) fairness perceptions with one item assessing one’s *team leader’s decision-making* (“We are treated fairly [by our team leader]”; *M* = 3.29, *SD* = 0.95). *Perceived diversity* was assessed with the same item as in Studies 1 and 2 (*M*  = 4.27, *SD* = 2.54). To create a dummy variable indicating whether team member and team leader belonged to the same ethnic group, we also queried *team leader group membership* (i.e., “Does your team leader belong to an ethnic minority group?” – 66 team members indicated to have an ethnic minority group member as their team leader). Finally, we also recorded whether participants were *permanently employed, temporarily employed with fixed term contract* or *temporarily employed with interim contract*, whether they were a *member* of the *union*, which *company site* they were working at and their *organizational* and *team tenure* (i.e., the number of years working in their current team).

# **Covariate Analyses**

**Sample 1a, Study 1**

The analyses revealed that organizational identification correlated significantly with participants’ age (*r*  = .12, *p*  = .001), interactional fairness (*r*  = .44, *p*  < .001), diversity beliefs (*r*  = .13, *p*  < .001) and perceived diversity (*r*  = -.17, *p*  < .001), whereas job satisfaction was significantly associated with interactional fairness (*r*  = .36, *p*  < .001), diversity beliefs (*r*  = .09, *p*  = .023) and perceived diversity (*r*  = -.14, *p*  < .001). Furthermore, it was found that organizational identification differed significantly between members of the union (*M*  = 3.96, *SD*  = 0.90) and non-members (*M*  = 4.21, *SD* = 0.72; *F*(1, 717) = 14.13, *p* = , *ŋ^2^* = .02), and between employees working day (*M*  = 4.27, *SD*  = 0.73) and night shifts (*M*  = 3.87, *SD*  = 1.05; *p* = .039). Moreover, results also revealed that job satisfaction differed significantly between participants who were employed fulltime (*M* = 4.13, *SD* = 0.85) and those who were employed part time (*M*  = 3.98, *SD* = 0.75; *F*(1, 533) = 7.08, *p* = .008, *ŋ^2^* = .01. As such, we retained all these variables in our main (mediation and moderation) analyses.

**Sample 1b, Study 1**

Our analyses revealed that organizational identification correlated significantly with diversity beliefs (*r*  = .12, *p* = .014), and that organizational identification differed significantly between members of the union (*M*  = 3.83, *SD*  = 0.79) and non-members (*M*  = 3.67, *SD* = 0.83; *F*(1, 435) = 3.88, *p* = .049, *ŋ^2^* = .01), and between employees working at the human resources department (*M*  = 3.60, *SD*  = 0.91) and employees working at the other company branch (*M*  = 3.80, *SD* = 0.81; *F*(1, 511) = 5.32, *p* = .021, *ŋ^2^* = .01).As such, we retained these covariates in our subsequent analyses.

**Sample 1c, Study 1**

Our analyses revealed that organizational identification was significantly related to organizational tenure (*r* = .17, *p* = .014), and that job satisfaction was significantly associated with Dutch language proficiency (*r* = .15, *p* = .029). As such, we retained both covariates in our main analyses.

**Study 2^[[2]](#footnote-2)^**

Our analyses revealed that organizational identification was significantly related to age (*r* = .25, *p* < .001) and income (*r* = .13, *p* = .030). Furthermore, organizational identification differed significantly between those with and without supervisory responsibilities (*M* = 3.42, *SD* = 0.66 and *M* = 3.17, *SD* = 0.78 respectively), those with and without experience being in a management position (*M* = 3.41, *SD* = 0.68 and *M* = 3.20, *SD* = 0.77), and those with and without decision-making responsibilities at work (*M* = 3.40, *SD* = 0.68 and *M* = 3.16, *SD* =0.80; all *F*s > 5.20, all *p*s < .024).

Secondly, our analyses revealed that job satisfaction was significantly related to age (*r* = .38, *p* < .001), education level (*r* = .32, *p* < .001), income (*r* = .30, *p* < .001), organizational tenure (*r* = .25, *p* < .001), the percentage of union members they estimated to be working in their organization (*r* = -.48, *p* < .001), the amount of hours worked per week (*r* = -.33, *p* < .001), participants’ company size (*r* = .47, *p* < .001), and the number of subordinates they had (*r* = -.46, *p* < .001).

Furthermore, organizational identification differed significantly between men and women (*M* = 3.59, *SD* = 1.14 and *M* = 2.68, *SD* = 1.51 respectively), those who were full-time and part-time employed (*M* = 2.92, *SD* = 1.46 and *M* = 3.74, *SD* = 1.08), between blue and white collar workers (*M* = 2.54, *SD* = 1.51 and *M* = 3.37, *SD* = 1.29), those with and without supervisory responsibilities (*M* = 2.93, *SD* = 1.48 and *M* = 3.30, *SD* = 1.32), those with and without experience being in a management position (*M* = 2.84, *SD* = 1.50 and *M* = 3.46, *SD* = 1.22), and those with and without decision-making responsibilities at work (*M* = 2.87, *SD* = 1.49 and *M* = 3.52, *SD* = 1.18; all *F*s > 4.35, all *p*s < .039). As such, we retained all the above variables in our main analyses.

**Study 3**

Our analyses revealed that organizational identification was significantly related to age (*r* = .10, *p* = .032), and that organizational identification differed significantly as a function of participants’ type of employment (*F*(2, 487) = 4.47, *p* = .012, *ŋ^2^* = .02): Those who were permanently employed (*M* = 3.53, *SD* = 0.90) reported lower organizational identification than those who were temporarily employed with an interim contract (*M* = 3.84, *SD* = 0.74, *p* = .009). Organizational identification did not differ between those who were permanently employed and those who were temporarily employed with a fixed term contract (*M*  = 3.62, *SD* = 0.78, *p* = .999), or between the two groups of temporarily employed workers (*p* = .733).

Furthermore, job satisfaction was significantly associated with education (*r* = -.11, *p* = .014), and job satisfaction differed significantly as a function of participants’ type of employment (*F*(2, 484) = 8.70, *p* < .001, *ŋ^2^* = .03): Those who were permanently employed (*M* = 3.60, *SD* = 0.96) reported lower job satisfaction than those who were temporarily employed with an interim contract (*M* = 4.07, *SD* = 0.76, *p* < .001). Job satisfaction did not differ between those who were permanently employed and those who were temporarily employed with a fixed term contract (*M*  = 3.61, *SD* = 0.99, *p* = .999), or between the two groups of temporarily employed workers (*p* = .059). As such, we retained these variables in our main analyses.

1. The item “In the course of the decision procedure, [company] does not show any preference for a specific group of employees” was dropped, because it could be interpreted as referring to one’s ethnic, cultural or linguistic group, and thus to EC decision making. [↑](#footnote-ref-1)
2. For the sake of brevity, only T1 covariate analyses are reported. [↑](#footnote-ref-2)
