# Supplementary material for: Ethnic-cultural procedural fairness effects on organizational identification and job satisfaction among minority and majority employees
Source: Front Psychol. 2025 Apr 15;16:1445469. doi: 10.3389/fpsyg.2025.1445469 (PMC12037562; doi:10.3389/fpsyg.2025.1445469)
Supplement: Supplementary file 2 [file Table_2.docx]

**Online Appendix**

This document contains the Online Appendix associated with the manuscript “ETHNIC-CULTURAL PROCEDURAL FAIRNESS EFFECTS ON ORGANIZATIONAL IDENTIFICATION AND JOB SATISFACTION AMONG MINORITY AND MAJORITY EMPLOYEES**”.**

Table A1 provides an exhaustive overview of demographic statistics and employee characteristics for all studies, and Table A2 schematically delineates all the study-specific covariates, and the items used to measure them. Tables B1-3 display the Study 1 (Samples 1a-1b-1c) correlations matrices, broken down by group membership (minority vs majority employees), Table B4 displays the Study 2 (T1 and T2) correlation matrices, and Table B5 display the Study 3 correlations matrices, broken down by group membership.

Tables C1 to C5 report the results of our Confirmatory Factor Analyses (CFAs) in Study 1 (Samples 1b and 1c), Study 2, and Study 3. Sections D1 and D2, finally, give a concise overview of our multilevel modeling procedures (Studies 2 and 3).

”,

**Section A: Sample descriptives and measures**

Table A1

*Descriptive Statistics and Employee Characteristics Per Sample*

| Study | *N*  *(% minority*) | Age: *M* (*SD*) | Gender (% males) | Education level (%) | | | | | Tenure org: *M* number of years (*SD*) |
| --- | --- | --- | --- | --- | --- | --- | --- | --- | --- |
|  |  |  |  | No degree | Primary edu | Secondary edu | Higher edu: Bachelor | Higher edu: Master or > |  |
| *Study 1, Sample 1a* | 801 (*26.6%)* | 36.98 (*10.63*) | 47.8 | 2.1 | 2.1 | 56.0 | 22.9 | 15.6 | 8.11 (*7.83*) |
| *Study 1, Sample 1b* | 514 *(15%)* | 41.68 *(10.27)* | 66.0 | 0.5 | 0.2 | 27.0 | 34.8 | 35.9 | 10.44 *(11.56)* |
| *Study 1, Sample 1c* | 235 *(28%)* | 45.36, (*10.08)* | 1.0 | 9.1 | 10.6 | 66.1 | 12.8 |  | 5.40 *(4.25)* |
| *Study 2* | 265 *(100%)* | 29.45  *(8.18)* | 43.8 |  | 34.3 | 41.9 | 21.1 | 2.6 | 3.55 *(4.29)* |
| *Study 3* | 509 *(35%)* | 20-30^1^ | 82.1 | 10.5 | 8.8 | 70.0 | 9.3 | 1.0 | 2.81 *(1.06)*/ 2.16 *(1.00)*^2^ |

*Note.* ^1^ This cell reflects the median age range in the sample. ^2^ Second number in cell reflects *M* team tenure (*SD* between brackets).

Table A1 (continued)

*Descriptive Statistics and Employee Characteristics Per Sample (continued)*

| Study | % union members | % permanently employed | % fulltime | Shift (*%*) | | | Blue vs White collar |
| --- | --- | --- | --- | --- | --- | --- | --- |
|  |  |  |  | Day | Night | Weekend | *(% Blue)* |
| *Study 1, Sample 1a* | 64.6 | 95.0 | 84.8 | 87.4 | 8.5 | 4.1 | *na* |
| *Study 1, Sample 1b* | 62.5 | *na* | *na* | *na* | *na* | *na* | 97.7 |
| *Study 1, Sample 1c* | *na* | *na* | *na* | *na* | *na* | *na* | 100 |
| *Study 2* | 44.2^1^ | *na* | 80.0 | 49.4 | 22.6 | *na* | 35.5 |
| *Study 3* | 90.0 | 76.3 | 6.3 | *na* | *na* | *na* | 100 |

*Note.* ^1^ This cell reflects the mean estimated percentage of union members working in participants’ respective organization.

Table A1 (continued)

*Descriptive Statistics and Employee Characteristics Per Sample (continued)*

| Study | Hours/week | Company size | Supervisor | Supervisor responsibilities | Number of subordinates | Management experience | Decision-making responsibilities |
| --- | --- | --- | --- | --- | --- | --- | --- |
|  |  |  |  |  |  |  |  |
| *Study 1, Sample 1a* |  |  |  |  |  |  |  |
| *Study 1, Sample 1b* |  |  |  |  |  |  |  |
| *Study 1, Sample 1c* |  |  |  |  |  |  |  |
| *Study 2* | 31-40^1^ *(49.8%)* | 1000+ ^1^ *(25.7%)* | Yes^1^ *(90.9%)* | Yes^1^  *(60.0%)* | 0-1^1^  *(43.8%)* | Yes^1^  *(61.5%)* | Yes^1^  *(68.3%)* |
| *Study 3* |  |  |  |  |  |  |  |

*Note.* ^1^ These cells reflects the response option that was most frequently selected by participants (selection frequency between parentheses).

Table A2

*Overview Of Study-Specific Covariates, Items Used to Measure Covariates, Means (SDs Between Brackets) and Reliabilities*

| Study | Variable | Items | *M* (*SD*) | *α* |
| --- | --- | --- | --- | --- |
| Study 1 |  |  |  |  |
| Sample 1a | Interactional fairness perceptions (with respect to EC decision-making) | “We are treated with respect” and “We are adequately informed” | 3.56 (0.92) | .72 |
|  | Perceived diversity | “How many members of your team belong to another ethnic-cultural group than yours?” ^1^ | 3.54 (2.25) |  |
|  | Diversity beliefs | “Is diversity in the workplace something that you value?” ^2^ | 4.09 (1.18) |  |
|  | Strength of religious affiliation | “How important is religion for you personally?” ^2^ | 2.62 (1.18) |  |
|  | Type of employment | “Are you temporarily or permanently employed?” |  |  |
|  | Type of contract | “Which contract do you have?” (fulltime/part-time) |  |  |
|  | Union membership | “Are you a member of the union?” (yes/no) |  |  |
|  | Shift | “Which shift are you currently working?” (day/night/weekend) |  |  |
|  | Company site | “In which company site are you employed?” (3 possibilities) |  |  |
|  | Organizational tenure | “How many years have you been working in [Company]? | 8.11 (7.83) |  |

*Notes.* Unless other response format is specified, items were scored on five-point Likert scales, ranging from 1 = “Totally disagree”, to 5 = “Totally agree”. ^1^: Anchored 1 = “None”, 10 = “All of them”. ^2^: Anchored 1 = “Not important at all”, 5 = “Very important”.

Table A2 (Continued)

| Study | Variable | Items | *M* (*SD*) | *α* |
| --- | --- | --- | --- | --- |
| Sample 1b | General procedural fairness | “We are able to voice our opinions”, “Accurate information is used to make decisions”, “We are given the opportunity to modify decisions”, “Decisions are made ethically”, and “Personal motives or biases influence decisions” (preceded by the sentence “In general, how are decisions made in [company]?”) | 2.75 (0.73) | .77 |
|  | Perceived diversity | “How many members of your team belong to another ethnic-cultural group than yours?” ^1^ | 1.82 (2.12) |  |
|  | Diversity beliefs | “Is diversity in the workplace something that you value?” ^2^ | 3.19 (1.11) |  |
|  | Strength of religious affiliation | “How important is religion for you personally?” ^2^ | 3.04 (1.11) |  |
|  | Perspectives on cultural diversity | “The presence of foreign cultures enriches our society”, “People belonging to different cultural groups should minimize their interactions” (Reverse-scored) and “There is not enough concern for people belonging to a different culture in [Country]” | 3.52 (0.85) | .71 |
|  | Union membership | “Are you a member of the union?” (yes/no) |  |  |
|  | Company branch | “In which company site are you employed?” (2 possibilities) |  |  |
|  | Type of labor | “Which type of work do you do?” (blue vs white collar work) |  |  |
|  | Organizational tenure | “How many years have you been working in [Company]? | 10.4 (11.56) |  |

Table A2 (Continued)

| Study | Variable | Items | *M* (*SD*) | *α* |
| --- | --- | --- | --- | --- |
| Sample 1c | General procedural fairness | “When my job coach makes decisions that concern me, he/she does it in a fair way” | 4.17 (0.80) |  |
|  | Dutch language proficiency | “How proficient are you in Dutch?” (1 = not proficient at all, 5 = very proficient) | 4.40 (0.93) |  |
| Study 2 |  |  |  |  |
|  | Employment status | What is your employment status? (full-time/part-time) |  |  |
|  | General procedural fairness | “I can count on my organization to have fair policies”, “My organization’s procedures and guidelines are very fair”, “My organization doesn’t have any fair policies” (R), “The procedures my organization uses to make decisions are not fair” (R) | 3.29 (0.95) |  |
|  | % union members | “Please estimate % union members working in your organization?” (0-100%) | 44.24 (37.58) |  |
|  | Type of labor | “Which type of work do you do?” (blue vs white collar work) |  |  |
|  | Shift | “What type of hours do you work?” (9-5/night shifts/rotating with night shift/rotating without night shift/other) |  |  |
|  | Hours/week | “How many hours do you work per week?” (1-10/11-20/21-30/31-40/41-50-51-60/>60) |  |  |
|  | Organizational tenure | “How many years have you worked for your employer? | 3.55 (4.29) |  |
|  | Company size | “How many employees in the company you work for?” (1-9/10-49/50-249/250-999/1000+) |  |  |

Table A2 (Continued)

| Study | Variable | Items | *M* (*SD*) | *α* |
| --- | --- | --- | --- | --- |
|  | Supervisor | “Do you have a direct supervisor?” (yes/no) |  |  |
|  | Supervisor responsibilities | “Do you have any supervisory responsibilities?” (yes/no) |  |  |
|  | Number of subordinates | “How many subordinates do you have?” (1/2-3/4-6/7-10/10+) |  |  |
|  | Management experience | “Do you have experience in management position?” (yes/no) |  |  |
|  | Decision-making responsibilities | “Do you have any decision-making responsibilities?”(yes/no) |  |  |
| Study 3 |  |  |  |  |
|  | Employment status | What is your employment status? (full-time/part-time/unemployed |  |  |
|  | General procedural fairness | “We are treated fairly [by our team leader]” | 3.29 (0.95) |  |
|  | Perceived diversity | “How many members of your team belong to another ethnic-cultural group than yours?” ^1^ | 4.27 (2.54) |  |
|  | Team leader group membership | “Is your team leader an ethnic minority member?” (yes/no) |  |  |
|  | Union membership | “Are you a member of the union?” (yes/no) |  |  |
|  | Type of employment | “Are you permanently, temporarily (with fixed term contract), or temporarily (with interim contract) employed?” |  |  |
|  | Company site | “In which company site are you employed?” (2 possibilities) |  |  |
|  | Team tenure | “How many years have you been working in your team?” | 2.16 (1.00) |  |
|  | Organizational tenure | “How many years have you been working in [Company]? | 10.4 (11.56) |  |

**Section B: Correlation matrices**

Table B1

*Correlations Among Key Sample 1a (Study 1) Variables, Broken Down by Group Membership (Minority vs Majority Group)*

|  | 1. | 2. | 3. | 4. | 5. | 6. | 7. | 8. |
| --- | --- | --- | --- | --- | --- | --- | --- | --- |
| 1. Procedural fairness (EC) |  | .41*** | .37*** | .65*** | -.24*** | .11* | .00 | -.12** |
| 2. Org. identification | .41*** |  | .50*** | .44*** | -.23*** | .13** | .03 | .04 |
| 3. Job satisfaction | .28*** | .57*** |  | .38*** | -.14** | .10* | .01 | -.05 |
| 4. Interactional fairness (EC) | .67*** | .42*** | .31*** |  | -.17*** | .11** | -.04 | -.03 |
| 5. Perceived diversity | -.04 | -.05 | -.11 | -.06 |  | -.03 | .15*** | -.03 |
| 6. Diversity beliefs | .02 | .15* | .09 | .04 | -.13† |  | .08† | .13** |
| 7. Strength religious aff. | -.12 | .04 | .05 | -.11 | .09 | .17* |  | .10* |
| 8. Org. tenure | -.22** | -.00 | -.07 | -.11 | -.10 | .09 | .01 |  |

*Notes. N* = 801. Correlations for minority group members are given below the diagonal, correlations for majority group members are presented above the diagonal. Org. identification = organizational identification. Strength religious aff. = strength of religious affiliation. Org. tenure = organizational tenure. †: *p* < .10. *: *p* < .05. **: *p*  < .01. ***: *p* < .001.

Table B2

*Correlations Among Key Sample 1b (Study 1) Variables, Broken Down by Group Membership (Minority vs Majority Group)*

|  | 1. | 2. | 3. | 4. | 5. | 6. | 7. | 8. | 9. |
| --- | --- | --- | --- | --- | --- | --- | --- | --- | --- |
| 1. Procedural fairness (EC) |  | .36*** | .36*** | .70*** | -.02 | .11* | .14** | -.01 | -.01 |
| 2. Org. identification | .42*** |  | .68*** | .41*** | -.01 | .13** | .01 | -.04 | .01 |
| 3. Job satisfaction | .48*** | .80*** |  | .46*** | -.09† | .03 | -.02 | -.06 | .02 |
| 4. Procedural fairness (Gen.) | .70*** | .41*** | .53*** |  | .03 | .16** | .13* | -.03 | .02 |
| 5. Perceived diversity | -.10 | -.17 | -.10 | -.00 |  | .08 | .01 | .07 | .00 |
| 6. Diversity beliefs | .22† | .03 | .05 | .21† | .15 |  | .53*** | .18*** | -.06 |
| 7. Diversity perspective | .02 | -.03 | -.06 | .04 | .07 | .44*** |  | .16** | -.12* |
| 8. Strength religious aff. | .04 | -.10 | .01 | .05 | .26* | .22† | .27* |  | .10† |
| 9. Org. tenure | -.03 | .09 | -.05 | -.05 | -.15 | -.08 | .23† | .05 |  |

*Notes. N* = 514. Correlations for minority group members are given below the diagonal, correlations for majority group members are presented above the diagonal. Org. identification = organizational identification. Strength religious aff. = strength of religious affiliation. Org. tenure = organizational tenure. †: *p* < .10. *: *p* < .05. **: *p*  < .01. ***: *p* < .001.

Table B3

*Correlations Among Key Sample 1c (Study 1) Variables, Broken Down by Group Membership (Minority vs Majority Group)*

|  | 1. | 2. | 3. | 4. | 5. | 6. |
| --- | --- | --- | --- | --- | --- | --- |
| 1. Procedural fairness (LP) |  | .18* | .02 | .36*** | .08 | .03 |
| 2. Org. identification | .71*** |  | .46*** | .40*** | .08 | .19* |
| 3. Job satisfaction | .38*** | .68*** |  | .27** | .06 | .09 |
| 4. Procedural fairness (JC) | .65*** | .74*** | .57*** |  | .05 | .12 |
| 5. Dutch language proficiency | .20 | -.11 | .03 | .16 |  | -.14† |
| 6. Org. tenure | .09 | .08 | -.06 | -.03 | .15 |  |

*Notes. N* = 235. Correlations for minority group members are given below the diagonal, correlations for majority group members are presented above the diagonal. Procedural fairness (LP) = procedural fairness perceptions with respect to language policy decision. Procedural fairness (JC) = procedural fairness perceptions with respect to job coach decision-making. Org. identification = organizational identification. Org. tenure = organizational tenure. †: *p* < .10. *: *p* < .05. **: *p*  < .01. ***: *p* < .001.

Table B4

*Correlations Among Key Study 2 Variables.*

|  | 1. | 2. | 3. | 4. | 5. | 6. |
| --- | --- | --- | --- | --- | --- | --- |
| 1. Procedural fairness (EC) |  | .57*** | .45*** | .58*** | -.10 | .09 |
| 2. Org. identification | .53*** |  | .74*** | .61*** | -.00 | .11 |
| 3. Job satisfaction | .22*** | .59*** |  | .59*** | -.02 | .09 |
| 4. Procedural fairness (Gen.) | .54*** | .53*** | .39*** |  | -.15 | .06 |
| 5. Income | .02 | .13* | .29*** | .01 |  | .09 |
| 6. Org. tenure | .04 | .18** | .24*** | .06 | .09 |  |

*Notes. N* = 265. T1 correlations are given below the diagonal, T2 correlations are presented above the diagonal. Procedural fairness (EC) = procedural fairness perceptions with respect to ethnic-cultural decision-making. Procedural fairness (gen.) = procedural fairness perceptions with respect to general team leader decision-making. Org. identification = organizational identification. Org. tenure = organizational tenure. *: *p* < .05. **: *p*  < .01. ***: *p* < .001.

Table B5

*Correlations Among Key Study 3 Variables, Broken Down by Group Membership (Minority vs Majority Group)*

|  | 1. | 2. | 3. | 4. | 5. | 6. | 7. |
| --- | --- | --- | --- | --- | --- | --- | --- |
| 1. Procedural fairness (EC) |  | .28*** | .30*** | .55*** | -.08 | -.12† | -.19** |
| 2. Org. identification | .47*** |  | .73*** | .39*** | -.04 | .08 | -.00 |
| 3. Job satisfaction | .43*** | .75*** |  | .44*** | -.04 | .02 | -.07 |
| 4. Procedural fairness (TL) | .66*** | .54*** | .52*** |  | -.09 | -.06 | -.19** |
| 5. Perceived diversity | .04 | .05 | .06 | .00 |  | -.13* | -.15* |
| 6. Org. tenure | -.23** | .01 | -.05 | -.21** | .07 |  | .54*** |
| 7. Team tenure | -.11 | .13† | .05 | .02 | .07 | .66 |  |

*Notes. N* = 509. Correlations for minority group members are given below the diagonal, correlations for majority group members are presented above the diagonal. Procedural fairness (EC) = procedural fairness perceptions with respect to ethnic-cultural decision-making. Procedural fairness (TL) = procedural fairness perceptions with respect to general team leader decision-making. Org. identification = organizational identification. Org. tenure = organizational tenure. †: *p* < .10. *: *p* < .05. **: *p*  < .01. ***: *p* < .001

**Section C: CFAs**

Table C1

*Results of CFA Sample 1b (study 1): Standardized factor loadings and item content*

| Item |  |  | Factor  I | Factor  II |
| --- | --- | --- | --- | --- |
| 1. We are able to voice our opinions. | | | .651 |  |
| 1. When making such decisions, [company] does not let personal biases or motives influence the decision process. | | | .555 |  |
| 1. We are given the opportunity to modify decisions that have already been made. | | | .735 |  |
| 1. Accurate information is used to make such decisions. | | | .739 |  |
| 1. Concern is shown for our rights. | | | .654 |  |
| 1. The input of various groups is obtained prior to making such decisions. | | | .771 |  |
| 1. We are able to voice our opinions. | | |  | .583 |
| 1. We are given the opportunity to modify decisions that have already been made, if we think that’s necessary. | | |  | .552 |
| 1. Accurate information is used to make such decisions. | | |  | .708 |
| 1. Concern is shown for our rights. | | |  | .718 |
| 1. The input of various groups is obtained prior to making such decisions. | | |  | .633 |

*Notes.* Factor I = EC procedural fairness perceptions. Factor II = general procedural fairness perceptions. Items 1-6 were preceded by the sentence “At [company], the workforce consists of people belonging to a variety of cultures, ethnicities and religions… And sometimes, decisions need to be made which affect an entire group of people belonging to the same ethnic-cultural group. Examples of such decisions are the decision to allow prayers at work, or the decision to customize the cafeteria menu in order to include kosher and/or halal food. How are, generally speaking, such decisions made according to you?”; items 7-11 were preceded by the sentence “In general, how are decisions made in [company]?”.

Table C2

*Results of CFA Sample 1c (Study 1): Standardized factor loadings and item content*

| Item |  |  | Factor  I | Factor  II |
| --- | --- | --- | --- | --- |
| 1. We are able to voice our opinions towards the “policy on language” of *[company]*. | | | .887 |  |
| 1. When making the decision about the “policy on language”, [company] did not let personal biases or motives influence the decision process. | | | .398 |  |
| 1. We were given the opportunity to modify the decision about the “policy on language” of [company]. | | | .359 |  |
| 1. Accurate information was used to make the decision about the “policy on language” of [company]. | | | .496 |  |
| 1. Concern was shown for our rights. | | | .433 |  |
| 1. The input of various groups was obtained prior to making the decision about the “policy on language” of [company]. | | | .658 |  |
| 1. When *my JOBCOACH* makes decisions that concern me, he does it in a fair way | | |  | 1.00 |

*Notes.* Factor I = EC procedural fairness perceptions. Factor II = general procedural fairness perceptions. Items 1-6 were preceded by the sentence “In [company] the management asks you to speak DUTCH AT WORK. The following statements are about this “LANGUAGE POLICY””; item 7 was preceded by the sentence “The following statements are about YOUR DAILY EXPERIENCES at [company]”.

Table C3

*Results of CFA Study 2 (T1): Standardized factor loadings and item content*

| Item |  |  | Factor  I | Factor  II |
| --- | --- | --- | --- | --- |
| 1. Consistent rules & procedures are used to make such decisions. | | | .517 |  |
| 1. Personal motives or biases influence such decisions. | | | .257 |  |
| 1. Such decisions are made ethically. | | | .775 |  |
| 1. Accurate information is used to make such decisions. | | | .705 |  |
| 1. Your input (and that of your ethnic, cultural or religious group) is obtained prior to making such decisions. | | | .613 |  |
| 1. You (and members of your ethnic, cultural or religious group) are given the opportunity to modify decisions that have already been made. | | | .588 |  |
| 1. The reasons behind these decisions are explained. | | | .704 |  |
| 1. Concern is shown for your (ethnic, cultural or religious) group's rights. | | | .682 |  |
| 1. There is a real interest in trying to be fair to you as a group. | | | .700 |  |
| 1. I can count on my organization to have fair policies. | | |  | .697 |
| 1. My oganization’s procedures and guidelines are very fair. | | |  | .836 |
| 1. My organization doesn’t have any fair policies. | | |  | .606 |
| 1. The procedures my organization uses to make decisions are not fair. | | |  | .723 |

*Notes.* Factor I = EC procedural fairness perceptions. Factor II = general procedural fairness perceptions. Items 1-9 were preceded by the sentence “At many companies, the workforce consists of people belonging to a variety of cultures, ethnicities and religions… And sometimes, decisions need to be made which affect an entire group of people belonging to the same ethnic, cultural, or religious group. Examples of such decision include: The decision to allow prayers at work, or the decision to customize the cafeteria menu in order to include kosher and/or halal food””.

Table C4

*Results of CFA Study 2 (T2): Standardized factor loadings and item content*

| Item |  |  | Factor  I | Factor  II |
| --- | --- | --- | --- | --- |
| 1. Consistent rules & procedures are used to make such decisions. | | | .692 |  |
| 1. Personal motives or biases influence such decisions. | | | .268 |  |
| 1. Such decisions are made ethically. | | | .735 |  |
| 1. Accurate information is used to make such decisions. | | | .755 |  |
| 1. Your input (and that of your ethnic, cultural or religious group) is obtained prior to making such decisions. | | | .674 |  |
| 1. You (and members of your ethnic, cultural or religious group) are given the opportunity to modify decisions that have already been made. | | | .723 |  |
| 1. The reasons behind these decisions are explained. | | | .833 |  |
| 1. Concern is shown for your (ethnic, cultural or religious) group's rights. | | | .761 |  |
| 1. There is a real interest in trying to be fair to you as a group. | | | .818 |  |
| 1. I can count on my organization to have fair policies. | | |  | .888 |
| 1. My oganization’s procedures and guidelines are very fair. | | |  | .861 |
| 1. My organization doesn’t have any fair policies. | | |  | .672 |
| 1. The procedures my organization uses to make decisions are not fair. | | |  | .665 |

*Notes.* Factor I = EC procedural fairness perceptions. Factor II = general procedural fairness perceptions.

Table C5

*Results of CFA Study 3: Standardized factor loadings and item content*

| Item |  |  | Factor  I | Factor  II |
| --- | --- | --- | --- | --- |
| 1. We are treated fairly when decisions are made that concern such cultural, ethnic and linguistic issues. | | | .768 |  |
| 1. We are treated respectfully when decisions are made that concern such cultural, ethnic and linguistic issues. | | | .844 |  |
| 1. We are able to voice our opinions when decisions are made that concern such cultural, ethnic and linguistic issues. | | | .859 |  |
| 1. We are being listened to when decisions are made that concern such cultural, ethnic and linguistic issues. | | | .889 |  |
| 1. We are treated fairly. | | |  | 1.00 |

*Notes.* Factor I = EC procedural fairness perceptions. Factor II = general procedural fairness perceptions. Items 1-4 were preceded by the sentence “At [company], the workforce consists of people belonging to a variety of cultures, ethnicities and religions… And sometimes, decisions need to be made which affect an entire group of people belonging to the same ethnic-cultural group. Examples of such decisions are the decision to allow prayers at work, or the decision to customize the cafeteria menu in order to include kosher and/or halal food. How are, generally speaking, such decisions made according to you?”; item 5 was preceded by the sentence “In what way are decisions generally made by your team supervisor?”.

**Section D: Multilevel modeling procedures**

## **Study 2**

Given the nested structure of our data (i.e., observations were nested within individuals^[[1]](#footnote-1)^), we first tested whether random intercepts were appropriate^[[2]](#footnote-2)^. Inspection of the ICC revealed that our ID (individual) clustering variable explained a large proportion of the variance in our hypothesized mediator (i.e., organizational identification; ICC = 50.60%) and a substantial proportion of the variance in our hypothesized outcome (i.e., job satisfaction; ICC = 15.90%). As such, we decided to add a random, individual-specific slope to our model. Thus, in our final model, individual measurement occasions were entered as the level-1 variable, and participants as the level-2 cluster variable. An overview of this final model is given in Table D1.

Table D1

*Results of multilevel modeling Study 2: Unstandardized parameter estimates for level-2 fixed effects, 95% confidence intervals [CIs], t values, and p-values*

| DV | Predictor | *b* (*SE*) | 95% CI | *t* | *p* |
| --- | --- | --- | --- | --- | --- |
| Organizational identification |  |  |  |  |  |
|  | **Procedural fairness** | **0.81 *(0.10)*** | **[0.61, 1.01]** | **7.89** | **<.001** |
|  | **Age** | **0.02 *(0.01)*** | **[0.01, 0.03]** | **3.07** | **.002** |
|  | Income | 0.01 *(0.02)* | [-0.02, 0.04] | 0.93 | .353 |
|  | Employment status: full-time | 0.03 *(0.09)* | [-0.14, 0.21] | 0.35 | .730 |
|  | Organizational tenure | 0.00 *(0.01)* | [-0.01, 0.02] | 0.36 | .717 |
|  | Supervisory responsibilities | 0.05 *(0.08)* | [-0.12, 0.21] | 0.59 | .557 |
|  | **Management experience** | **-0.23 *(0.08)*** | **[-0.39, -0.07]** | **-2.75** | **.006** |
|  | Decision-making responsibilities | 0.15 *(0.09)* | [-0.02, 0.32] | 1.73 | .084 |
|  |  |  |  |  |  |
| Job satisfaction |  |  |  |  |  |
|  | **Procedural fairness** | **0.68 *(0.26)*** | **[0.17, 1.19]** | **2.59** | **.010** |
|  | **Organizational identification** | **0.66 *(0.20)*** | **[0.26, 1.06]** | **3.25** | **.001** |
|  | Age | 0.01 *(0.01)* | [-0.00, 0.02] | 1.40 | .161 |
|  | Gender | 0.03 *(0.09)* | [-0.14, 0.21] | 0.38 | .704 |
|  | Education | -0.05 *(0.06)* | [-0.16, 0.06] | -0.94 | .347 |
|  | Income | 0.02 *(0.02)* | [-0.02, 0.05] | 0.73 | .465 |
|  | **% union members** | **-0.00 *(0.00)*** | **[-0.01, -0.00]** | **-2.14** | **.033** |
|  | Employment status: full-time | -0.08 *(0.15)* | [-0.37, 0.22] | -0.51 | .611 |
|  | Organizational tenure | 0.01 *(0.01)* | [-0.01, 0.03] | 1.06 | .287 |
|  | **Type of labor** | **-0.21 *(0.12)*** | **[-0.39, -0.03]** | **-2.27** | **.023** |
|  | Hours/week | -0.03 *(0.09)* | [-0.12, 0.06] | -0.63 | .526 |
|  | Company size | 0.06 *(0.03)* | [-0.01, 0.12] | 1.76 | .079 |
|  | Supervisory responsibilities | 0.09 *(0.12)* | [-0.15, 0.33] | 0.75 | .452 |
|  | Management experience | -0.23 *(0.12)* | [-0.46, 0.01] | -1.88 | .060 |
|  | **Number of subordinates** | **-0.11 *(0.03)*** | **[-0.18, -0.04]** | **-3.15** | **.002** |
|  | Decision-making responsibilities | -0.00 *(0.11)* | [-0.22, 0.22] | -0.02 | .983 |
|  | Shift: 9-5 | 0.27 *(0.26)* | [-0.25, 0.78] | 1.02 | .309 |
|  | Shift: Night shifts | 0.19 *(0.16)* | [-0.12, 0.51] | 1.20 | .230 |
|  | Shift: Rotating with night shift | -0.06 *(0.13)* | [-0.32, 0.20] | -0.46 | .644 |
|  | Shift: Rotating without night shifts | -0.10 *(0.13)* | [-0.34, 0.15] | -0.79 | .429 |

| *Note. N* = 265. Employment status: 1 = full-time, 0 = part-time. Supervisory responsibilities: 1 = yes, 0 = no. Management experience: 1 = yes, 0 = no. Decision-making responsibilities: 1 = yes, 0 = no. Type of labor: 1 = blue-collar, 0 = white-collar. Shift: 1 = 9-5/night shifts/rotating with night shift/rotating without night shift, 0 = other. |
| --- |

## **Study 3**

Given the nested structure of our data (i.e., individuals were nested within teams), we first tested whether a random intercept and a random slope were necessary. Although inspection of the ICC revealed that our team clustering variable explained only small proportions of the variance in our hypothesized mediator (i.e., organizational identification; ICC = 1.30%) and our hypothesized outcome (i.e., job satisfaction; ICC = 2.30%), we nonetheless adopted a conservative approach and added a random, team-specific intercept to our model^[[3]](#footnote-3)^. The results of a likelihood ratio test further revealed that a model including random slopes did not significantly outperform a model without random slopes (AIC(random-slopes model) = 1004.26, AIC(random-intercept model) = 997.66; *χ*^2^(5) = 3.40, *p* = .639). As such, we fitted a random-intercept mediation model only.

As before, we first attempted to fit the simple multilevel mediation model for the minority group subsample. This model, however, did not converge, due to the small number of observations per team cluster (max *n* = 9, *M* = 2.71). As such, we decided to move on and fit the multilevel (moderated) mediation model on the full sample. An overview of this final model is given in Table D2.

Table D2

*Results of multilevel modeling Study 3: Unstandardized parameter estimates for level-1 fixed effects, 95% confidence intervals [CIs], t values, and p-values*

| DV | Predictor | *b* (*SE*) | 95% CI | *t* | *p* |
| --- | --- | --- | --- | --- | --- |
| Organizational identification |  |  |  |  |  |
|  | **Procedural fairness** | **0.21 *(0.05)*** | **[0.11, 0.30]** | **4.23** | **<.001** |
|  | Group membership | 0.04 *(0.16)* | [-0.29, 0.37] | 0.24 | .810 |
|  | PF*Group membership | -0.05 *(0.05)* | [-0.15, 0.05] | -1.00 | .331 |
|  | **Age** | **0.09 *(0.04)*** | **[0.01, 0.18]** | **2.82** | **.025** |
|  | Type of employment: permanent | -0.20 *(0.11)* | [-0.42, 0.02] | -0.62 | .081 |
|  | Type of employment: temporary (F) | -0.12 *(0.19)* | [-0.49, 0.25] | -1.75 | .534 |
|  |  |  |  |  |  |
| Job satisfaction |  |  |  |  |  |
|  | Procedural fairness | 0.02 *(0.04)* | [-0.05, 0.10] | 0.66 | .514 |
|  | **Organizational identification** | **0.78 *(0.04)*** | **[0.70, 0.85]** | **19.46** | **<.001** |
|  | PF*Group membership | 0.01 *(0.02)* | [-0.03, 0.05] | 0.34 | .736 |
|  | Education | -0.05 *(0.04)* | [-0.13, 0.03] | -1.27 | .206 |
|  | Type of employment: permanent | -0.15 *(0.09)* | [-0.31, 0.02] | -1.74 | .083 |
|  | Type of employment: temporary (F) | -0.28 *(0.15)* | [-0.56, 0.01] | -1.92 | .055 |

*Note. N* = 509. Type of employment (permanent, temporary [F = fixed term contract], or temporary [I = interim contract]) was dummy-coded, reference level 0 = temporary [I].

1. We acknowledge that participants were also nested within countries As such, we first attempted to fit a multilevel mediation model with repeated measurement occasions as level-1 variable, participants as level-2 cluster variable, and country as level-3 cluster variable. This model, however, did not converge, due to the small number of observations in some countries. [↑](#footnote-ref-1)
2. Note that we did not include random slopes, because the number of random effects would have equaled the number of observations, rendering the residual variance unidentifiable. [↑](#footnote-ref-2)
3. Note that our results were virtually identical when we fitted a linear mediation model (i.e., without a random, team-specific intercept). [↑](#footnote-ref-3)
